# Comparative effectiveness and safety of non-vitamin K antagonists for atrial fibrillation in clinical practice: GLORIA-AF Registry

**DOI:** 10.1007/s00392-022-01996-2

**Published:** 2022-03-16

**Authors:** Gregory Y. H. Lip, Agnieszka Kotalczyk, Christine Teutsch, Hans-Christoph Diener, Sergio J. Dubner, Jonathan L. Halperin, Chang-Sheng Ma, Kenneth J. Rothman, Sabrina Marler, Venkatesh Kumar Gurusamy, Menno V. Huisman, for the GLORIA-AF Investigators, Dzifa Wosornu Abban, Emad Aziz, Marica Bracic Kalan, Nasser Abdul, Luciano Marcelo Backes, Drew Bradman, Atilio Marcelo Abud, E. Badings, Donald Brautigam, Fran Adams, Ermentina Bagni, Nicolas Breton, Srinivas Addala, Seth H. Baker, P. J. A. M. Brouwers, Pedro Adragão, Richard Bala, Kevin Browne, Walter Ageno, Antonio Baldi, Jordi Bruguera Cortada, Rajesh Aggarwal, Shigenobu Bando, A. Bruni, Sergio Agosti, Subhash Banerjee, Claude Brunschwig, Piergiuseppe Agostoni, Alan Bank, Hervé Buathier, Francisco Aguilar, Gonzalo Barón Esquivias, Aurélie Buhl, Julio Aguilar Linares, Craig Barr, John Bullinga, Luis Aguinaga, Maria Bartlett, Jose Walter Cabrera, Jameel Ahmed, Vanja Basic Kes, Alberto Caccavo, Allessandro Aiello, Giovanni Baula, Shanglang Cai, Paul Ainsworth, Steffen Behrens, Sarah Caine, Jorge Roberto Aiub, Alan Bell, Leonardo Calò, Raed Al-Dallow, Raffaella Benedetti, Valeria Calvi, Lisa Alderson, Juan Benezet Mazuecos, Mauricio Camarillo Sánchez, Jorge Antonio Aldrete Velasco, Bouziane Benhalima, Rui Candeias, Dimitrios Alexopoulos, Jutta Bergler-Klein, Vincenzo Capuano, Fernando Alfonso Manterola, Jean-Baptiste Berneau, Alessandro Capucci, Pareed Aliyar, Richard A. Bernstein, Ronald Caputo, David Alonso, Percy Berrospi, Tatiana Cárdenas Rizo, Fernando Augusto Alves da Costa, Sergio Berti, Francisco Cardona, José Amado, Andrea Berz, Francisco Carlos da Costa Darrieux, Walid Amara, Elizabeth Best, Yan Carlos Duarte Vera, Mathieu Amelot, Paulo Bettencourt, Antonio Carolei, Nima Amjadi, Robert Betzu, Susana Carreño, Fabrizio Ammirati, Ravi Bhagwat, Paula Carvalho, Marianna Andrade, Luna Bhatta, Susanna Cary, Nabil Andrawis, Francesco Biscione, Gavino Casu, Giorgio Annoni, Giovanni Bisignani, Claudio Cavallini, Gerardo Ansalone, Toby Black, Guillaume Cayla, M. Kevin Ariani, Michael J. Bloch, Aldo Celentano, Juan Carlos Arias, Stephen Bloom, Tae-Joon Cha, Sébastien Armero, Edwin Blumberg, Kwang Soo Cha, Chander Arora, Mario Bo, Jei Keon Chae, Muhammad Shakil Aslam, Ellen Bøhmer, Kathrine Chalamidas, M. Asselman, Andreas Bollmann, Krishnan Challappa, Philippe Audouin, Maria Grazia Bongiorni, Sunil Prakash Chand, Charles Augenbraun, Giuseppe Boriani, Harinath Chandrashekar, S. Aydin, D. J. Boswijk, Ludovic Chartier, S. Aydin, Jochen Bott, Kausik Chatterjee, Ivaneta Ayryanova, Edo Bottacchi, Carlos Antero Chavez Ayala, Aamir Cheema, Gershan Davis, Rudolph Evonich, Amjad Cheema, Jean-Marc Davy, Oksana Evseeva, Lin Chen, Mark Dayer, Andrey Ezhov, Shih-Ann Chen, Marzia De Biasio, Raed Fahmy, Jyh Hong Chen, Silvana De Bonis, Quan Fang, Fu-Tien Chiang, Raffaele De Caterina, Ramin Farsad, Francesco Chiarella, Teresiano De Franceschi, Laurent Fauchier, Lin Chih-Chan, J. R. de Groot, Stefano Favale, Yong Keun Cho, José De Horta, Maxime Fayard, Jong-Il Choi, Axel De La Briolle, Jose Luis Fedele, Dong Ju Choi, Gilberto de la Pena Topete, Francesco Fedele, Guy Chouinard, Angelo Amato Vicenzo de Paola, Olga Fedorishina, Danny Hoi-Fan Chow, Weimar de Souza, Steven R. Fera, Dimitrios Chrysos, A. de Veer, Luis Gustavo Gomes Ferreira, Galina Chumakova, Luc De Wolf, Jorge Ferreira, Eduardo Julián José Roberto Chuquiure Valenzuela, Eric Decoulx, Claudio Ferri, Nicoleta Cindea Nica, Sasalu Deepak, Anna Ferrier, David J. Cislowski, Pascal Defaye, Hugo Ferro, Anthony Clay, Freddy Del-Carpio Munoz, Alexandra Finsen, Piers Clifford, Diana Delic Brkljacic, Brian First, Andrew Cohen, N. Joseph Deumite, Stuart Fischer, Michael Cohen, Silvia Di Legge, Catarina Fonseca, Serge Cohen, Igor Diemberger, Luísa Fonseca Almeida, Furio Colivicchi, Denise Dietz, Steven Forman, Ronan Collins, Pedro Dionísio, Brad Frandsen, Paolo Colonna, Qiang Dong, William French, Steve Compton, Fabio Rossi dos Santos, Keith Friedman, Derek Connolly, Elena Dotcheva, Athena Friese, Alberto Conti, Rami Doukky, Ana Gabriela Fruntelata, Gabriel Contreras Buenostro, Anthony D’Souza, Shigeru Fujii, Gregg Coodley, Simon Dubrey, Stefano Fumagalli, Martin Cooper, Xavier Ducrocq, Marta Fundamenski, Julian Coronel, Dmitry Dupljakov, Yutaka Furukawa, Giovanni Corso, Mauricio Duque, Matthias Gabelmann, Juan Cosín Sales, Dipankar Dutta, Nashwa Gabra, Yves Cottin, Nathalie Duvilla, Niels Gadsbøll, John Covalesky, A. Duygun, Michel Galinier, Aurel Cracan, Rainer Dziewas, Anders Gammelgaard, Filippo Crea, Charles B. Eaton, Priya Ganeshkumar, Peter Crean, William Eaves, Christopher Gans, James Crenshaw, L. A. Ebels-Tuinbeek, Antonio Garcia Quintana, Tina Cullen, Clifford Ehrlich, Olivier Gartenlaub, Harald Darius, Sabine Eichinger-Hasenauer, Achille Gaspardone, Patrick Dary, Steven J. Eisenberg, Conrad Genz, Olivier Dascotte, Adnan El Jabali, Frédéric Georger, Ira Dauber, Mahfouz El Shahawy, Jean-Louis Georges, Vicente Davalos, Mauro Esteves Hernandes, Steven Georgeson, Ruth Davies, Ana Etxeberria Izal, Evaldas Giedrimas, Mariusz Gierba, Tetsuya Haruna, Nabil Jarmukli, Ignacio Gil Ortega, Emil Hayek, Robert J. Jeanfreau, Eve Gillespie, Jeff Healey, Ronald D. Jenkins, Alberto Giniger, Steven Hearne, Carlos Jerjes Sánchez, Michael C. Giudici, Michael Heffernan, Javier Jimenez, Alexandros Gkotsis, Geir Heggelund, Robert Jobe, Taya V. Glotzer, J. A. Heijmeriks, Tomas Joen-Jakobsen, Joachim Gmehling, Maarten Hemels, Nicholas Jones, Jacek Gniot, I. Hendriks, Jose Carlos Moura Jorge, Peter Goethals, Sam Henein, Bernard Jouve, Seth Goldbarg, Sung-Ho Her, Byung Chun Jung, Ronald Goldberg, Paul Hermany, Kyung Tae Jung, Britta Goldmann, Jorge Eduardo Hernández Del Río, Werner Jung, Sergey Golitsyn, Yorihiko Higashino, Mikhail Kachkovskiy, Silvia Gómez, Michael Hill, Krystallenia Kafkala, Juan Gomez Mesa, Tetsuo Hisadome, Larisa Kalinina, Vicente Bertomeu Gonzalez, Eiji Hishida, Bernd Kallmünzer, Jesus Antonio Gonzalez Hermosillo, Etienne Hoffer, Farzan Kamali, Víctor Manuel González López, Matthew Hoghton, Takehiro Kamo, Hervé Gorka, Kui Hong, Priit Kampus, Charles Gornick, Suk keun Hong, Hisham Kashou, Diana Gorog, Stevie Horbach, Andreas Kastrup, Venkat Gottipaty, Masataka Horiuchi, Apostolos Katsivas, Pascal Goube, Yinglong Hou, Elizabeth Kaufman, Ioannis Goudevenos, Jeff Hsing, Kazuya Kawai, Brett Graham, Chi-Hung Huang, Kenji Kawajiri, G. Stephen Greer, David Huckins, John F. Kazmierski, Uwe Gremmler, Kathy Hughes, P. Keeling, Paul G. Grena, A. Huizinga, José Francisco Kerr Saraiva, Martin Grond, E. L. Hulsman, Galina Ketova, Edoardo Gronda, Kuo-Chun Hung, AJIT Singh Khaira, Gerian Grönefeld, Gyo-Seung Hwang, Aleksey Khripun, Xiang Gu, Margaret Ikpoh, Doo-Il Kim, Ivett Guadalupe Torres Torres, Davide Imberti, Young Hoon Kim, Gabriele Guardigli, Hüseyin Ince, Nam Ho Kim, Carolina Guevara, Ciro Indolfi, Dae Kyeong Kim, Alexandre Guignier, Shujiro Inoue, Jeong Su Kim, Michele Gulizia, Didier Irles, June Soo Kim, Michael Gumbley, Harukazu Iseki, Ki Seok Kim, Albrecht Günther, C. Noah Israel, Jin bae Kim, Andrew Ha, Bruce Iteld, Elena Kinova, Georgios Hahalis, Venkat Iyer, Alexander Klein, Joseph Hakas, Ewart Jackson-Voyzey, James J. Kmetzo, Christian Hall, Naseem Jaffrani, G. Larsen Kneller, Bing Han, Frank Jäger, Aleksandar Knezevic, Seongwook Han, Martin James, Su Mei Angela Koh, Joe Hargrove, Sung-Won Jang, Shunichi Koide, David Hargroves, Nicolas Jaramillo, Anastasios Kollias, J. A. Kooistra, Weihua Li, John McClure, Jay Koons, Xiaoming Li, Terry McCormack, Martin Koschutnik, Christhoh Lichy, William McGarity, William J. Kostis, Ira Lieber, Hugh McIntyre, Dragan Kovacic, Ramon Horacio Limon Rodriguez, Brent McLaurin, Jacek Kowalczyk, Hailong Lin, Feliz Alvaro, Medina Palomino, Natalya Koziolova, Gregory Y. H. Lip, Francesco Melandri, Peter Kraft, Feng Liu, Hiroshi Meno, Johannes A. Kragten, Hengliang Liu, Dhananjai Menzies, Mori Krantz, Guillermo Llamas Esperon, Marco Mercader, Lars Krause, Nassip Llerena Navarro, Christian Meyer, B. J. Krenning, Eric Lo, Beat J. Meyer, F. Krikke, Sergiy Lokshyn, Jacek Miarka, Z. Kromhout, Amador López, Frank Mibach, Waldemar Krysiak, José Luís López-Sendón, Dominik Michalski, Priya Kumar, Adalberto Menezes Lorga Filho, Patrik Michel, Thomas Kümler, Richard S. Lorraine, Rami Mihail Chreih, Malte Kuniss, Carlos Alberto Luengas, Alberto Luengas, Ghiath Mikdadi, Jen-Yuan Kuo, Robert Luke, Milan Mikus, Achim Küppers, Ming Luo, Davor Milicic, Karla Kurrelmeyer, Steven Lupovitch, Constantin Militaru, Choong Hwan Kwak, Philippe Lyrer, Sedi Minaie, Bénédicte Laboulle, Changsheng Ma, Bogdan Minescu, Arthur Labovitz, Genshan Ma, Iveta Mintale, Wen Ter Lai, Irene Madariaga, Tristan Mirault, Andy Lam, Koji Maeno, Michael J. Mirro, Yat Yin Lam, Dominique Magnin, Dinesh Mistry, Fernando Lanas Zanetti, Gustavo Maid, Nicoleta Violeta Miu, Charles Landau, Sumeet K. Mainigi, Naomasa Miyamoto, Giancarlo Landini, Konstantinos Makaritsis, Tiziano Moccetti, Estêvão Lanna Figueiredo, Rohit Malhotra, Akber Mohammed, Torben Larsen, Rickey Manning, Azlisham Mohd Nor, Karine Lavandier, Athanasios Manolis, Michael Mollerus, Jessica LeBlanc, Helard Andres Manrique Hurtado, Giulio Molon, Moon Hyoung Lee, Ioannis Mantas, Sergio Mondillo, Chang-Hoon Lee, Fernando Manzur Jattin, Patrícia Moniz, John Lehman, Vicky Maqueda, Lluis Mont, Ana Leitão, Niccolo Marchionni, Vicente Montagud, Nicolas Lellouche, Francisco Marin Ortuno, Oscar Montaña, Malgorzata Lelonek, Antonio Martín Santana, Cristina Monti, Radoslaw Lenarczyk, Jorge Martinez, Luciano Moretti, T. Lenderink, Petra Maskova, Kiyoo Mori, Salvador León González, Norberto Matadamas Hernandez, Andrew Moriarty, Peter Leong-Sit, Katsuhiro Matsuda, Jacek Morka, Matthias Leschke, Tillmann Maurer, Luigi Moschini, Nicolas Ley, Ciro Mauro, Nikitas Moschos, Zhanquan Li, Erik May, Andreas Mügge, Xiaodong Li, Nolan Mayer, Thomas J. Mulhearn, Carmen Muresan, Eena Padayattil Jose, Dalton Bertolim Précoma, Michela Muriago, Francisco Gerardo Padilla Padilla, Alessandro Prelle, Wlodzimierz Musial, Victoria Padilla Rios, John Prodafikas, Carl W. Musser, Giuseppe Pajes, Konstantin Protasov, Francesco Musumeci, A. Shekhar Pandey, Maurice Pye, Thuraia Nageh, Gaetano Paparella, Zhaohui Qiu, Hidemitsu Nakagawa, F. Paris, Jean-Michel Quedillac, Yuichiro Nakamura, Hyung Wook Park, Dimitar Raev, Toru Nakayama, Jong Sung Park, Carlos Antonio Raffo Grado, Gi-Byoung Nam, Fragkiskos Parthenakis, Sidiqullah Rahimi, Michele Nanna, Enrico Passamonti, Arturo Raisaro, Indira Natarajan, Rajesh J. Patel, Bhola Rama, Hemal M. Nayak, Jaydutt Patel, Ricardo Ramos, Stefan Naydenov, Mehool Patel, Maria Ranieri, Jurica Nazlić, Janice Patrick, Nuno Raposo, Alexandru Cristian Nechita, Ricardo Pavón Jimenez, Eric Rashba, Libor Nechvatal, Analía Paz, Ursula Rauch-Kroehnert, Sandra Adela Negron, Vittorio Pengo, Ramakota Reddy, James Neiman, William Pentz, Giulia Renda, Fernando Carvalho Neuenschwander, Beatriz Pérez, Shabbir Reza, David Neves, Alma Minerva Pérez Ríos, Luigi Ria, Anna Neykova, Alejandro Pérez-Cabezas, Dimitrios Richter, Ricardo Nicolás Miguel, Richard Perlman, Hans Rickli, George Nijmeh, Viktor Persic, Werner Rieker, Alexey Nizov, Francesco Perticone, Tomas Ripolil Vera, Rodrigo Noronha Campos, Terri K. Peters, Luiz Eduardo Ritt, Janko Nossan, Sanjiv Petkar, Douglas Roberts, Tatiana Novikova, Luis Felipe Pezo, Ignacio Rodriguez Briones, Ewa Nowalany-Kozielska, Christian Pflücke, Aldo Edwin Rodriguez Escudero, Emmanuel Nsah, David N. Pham, Carlos Rodríguez Pascual, Juan Carlos Nunez Fragoso, Roland T. Phillips, Mark Roman, Svetlana Nurgalieva, Stephen Phlaum, Francesco Romeo, Dieter Nuyens, Denis Pieters, E. Ronner, Ole Nyvad, Julien Pineau, Jean-Francois Roux, Manuel Odin de Los Rios Ibarra, Arnold Pinter, Nadezda Rozkova, Philip O’Donnell, Fausto Pinto, Miroslav Rubacek, Martin O’Donnell, R. Pisters, Frank Rubalcava, Seil Oh, Nediljko Pivac, Andrea M. Russo, Yong Seog Oh, Darko Pocanic, Matthieu Pierre Rutgers, Dongjin Oh, Cristian Podoleanu, Karin Rybak, Gilles O‘Hara, Alessandro Politano, Samir Said, Kostas Oikonomou, Zdravka Poljakovic, Tamotsu Sakamoto, Claudia Olivares, Stewart Pollock, Abraham Salacata, Richard Oliver, Jose Polo Garcéa, Adrien Salem, Rafael Olvera Ruiz, Holger Poppert, Rafael Salguero Bodes, Christoforos Olympios, Maurizio Porcu, Marco A. Saltzman, Anna Omaszuk-Kazberuk, Antonio Pose Reino, Alessandro Salvioni, Joaquín Osca Asensi, Neeraj Prasad, Gregorio Sanchez Vallejo, Marcelo Sanmartín Fernández, Adam Sokal, Tian Ming Tu, Wladmir Faustino Saporito, Yannie Soo Oi Yan, Ype Tuininga, Kesari Sarikonda, Rodolfo Sotolongo, Minang Turakhia, Taishi Sasaoka, Olga Ferreira de Souza, Samir Turk, Hamdi Sati, Jon Arne Sparby, Wayne Turner, Irina Savelieva, Jindrich Spinar, Arnljot Tveit, Pierre-Jean Scala, David Sprigings, Richard Tytus, Peter Schellinger, Alex C. Spyropoulos, C. Valadão, Carlos Scherr, Dimitrios Stakos, P. F. M. M. van Bergen, Lisa Schmitz, Clemens Steinwender, Philippe van de Borne, Karl-Heinz Schmitz, Georgios Stergiou, B. J. van den Berg, Bettina Schmitz, Ian Stiell, C. van der Zwaan, Teresa Schnabel, Marcus Stoddard, M. Van Eck, Steffen Schnupp, Anastas Stoikov, Peter Vanacker, Peter Schoeniger, Witold Streb, Dimo Vasilev, Norbert Schön, Ioannis Styliadis, Vasileios Vasilikos, Peter Schwimmbeck, Guohai Su, Maxim Vasilyev, Clare Seamark, Xi Su, Srikar Veerareddy, Greg Searles, Wanda Sudnik, Mario Vega Miño, Karl-Heinz Seidl, Kai Sukles, Asok Venkataraman, Barry Seidman, Xiaofei Sun, Paolo Verdecchia, Jaroslaw Sek, H. Swart, Francesco Versaci, Lakshmanan Sekaran, Janko Szavits-Nossan, Ernst Günter Vester, Carlo Serrati, Jens Taggeselle, Hubert Vial, Neerav Shah, Yuichiro Takagi, Jason Victory, Vinay Shah, Amrit Pal Singh Takhar, Alejandro Villamil, Anil Shah, Angelika Tamm, Marc Vincent, Shujahat Shah, Katsumi Tanaka, Anthony Vlastaris, Vijay Kumar Sharma, Tanyanan Tanawuttiwat, Jürgen vom Dahl, Louise Shaw, Sherman Tang, Kishor Vora, Khalid H. Sheikh, Aylmer Tang, Robert B. Vranian, Naruhito Shimizu, Giovanni Tarsi, Paul Wakefield, Hideki Shimomura, Tiziana Tassinari, Ningfu Wang, Dong-Gu Shin, Ashis Tayal, Mingsheng Wang, Eun-Seok Shin, Muzahir Tayebjee, Xinhua Wang, Junya Shite, J. M. ten Berg, Feng Wang, Gerolamo Sibilio, Dan Tesloianu, Tian Wang, Frank Silver, Salem H. K. The, Alberta L. Warner, Iveta Sime, Dierk Thomas, Kouki Watanabe, Tim A. Simmers, Serge Timsit, Jeanne Wei, Narendra Singh, Tetsuya Tobaru, Christian Weimar, Peter Siostrzonek, Andrzej R. Tomasik., Stanislav Weiner, Didier Smadja, Mikhail Torosoff, Renate Weinrich, David W. Smith, Emmanuel Touze, Ming-Shien Wen, Marcelo Snitman, Elina Trendafilova, Marcus Wiemer, Dario Sobral Filho, W. Kevin Tsai, Preben Wiggers, Hassan Soda, Hung Fat Tse, Andreas Wilke, Carl Sofley, Hiroshi Tsutsui, David Williams, Marcus L. Williams, Ping Yen Bryan Yan, Ping Zhang, Bernhard Witzenbichler, Tianlun Yang, Jun Zhang, Brian Wong, Jing Yao, Shui Ping Zhao, Ka Sing Lawrence Wong, Kuo-Ho Yeh, Yujie Zhao, Beata Wozakowska-Kaplon, Wei Hsian Yin, Zhichen Zhao, Shulin Wu, Yoto Yotov, Yang Zheng, Richard C. Wu, Ralf Zahn, Jing Zhou, Silke Wunderlich, Stuart Zarich, Sergio Zimmermann, Nell Wyatt, Sergei Zenin, Andrea Zini, John Wylie, Elisabeth Louise Zeuthen, Steven Zizzo, Yong Xu, Huanyi Zhang, Wenxia Zong, Xiangdong Xu, Donghui Zhang, LSteven Zukerman, Hiroki Yamanoue, Xingwei Zhang, Takeshi Yamashita

**Affiliations:** 1 Liverpool Centre for Cardiovascular Science, University of Liverpool and Liverpool Heart and Chest Hospital, Liverpool, UK; 2 Department of Cardiology, Congenital Heart Diseases and Electrotherapy, Medical University of Silesia, Silesian Centre for Heart Diseases, Zabrze, Poland; 3 Aalborg Thrombosis Research Unit, Department of Clinical Medicine, Aalborg University, Aalborg, Denmark; 4 Department of Cardiometabolism and Respiratory Medicine, Boehringer Ingelheim International GmbH, Ingelheim, Germany; 5 Department of Neuroepidemiology, Institute for Medical Informatics, Biometry and Epidemiology (IMIBE), University Duisburg-Essen, Essen, Germany; 6 Clínica y Maternidad Suizo Argentina, Buenos Aires, Argentina; 7 The Cardiovascular Institute, Mount Sinai Medical Center, New York, NY USA; 8 Cardiology Department, Atrial Fibrillation Center, Beijing Anzhen Hospital, Capital Medical University, Beijing, China; 9 RTI Health Solutions, Research Triangle Park, NC USA; 10 Biostatistics and Data Sciences, Boehringer Ingelheim Pharmaceuticals, Inc, Ridgefield, CT USA; 11 Global Epidemiology, Boehringer Ingelheim International GmbH, Ingelheim, Germany; 12 Department of Thrombosis and Hemostasis, Leiden University Medical Center, Leiden, The Netherlands

**Keywords:** Atrial fibrillation, Non-vitamin K antagonists, Dabigatran, Rivaroxaban, Apixaban

## Abstract

**Background and purpose:**

Prospectively collected data comparing the safety and effectiveness of individual non-vitamin K antagonists (NOACs) are lacking. Our objective was to directly compare the effectiveness and safety of NOACs in patients with newly diagnosed atrial fibrillation (AF).

**Methods:**

In GLORIA-AF, a large, prospective, global registry program, consecutive patients with newly diagnosed AF were followed for 3 years. The comparative analyses for (1) dabigatran vs rivaroxaban or apixaban and (2) rivaroxaban vs apixaban were performed on propensity score (PS)-matched patient sets. Proportional hazards regression was used to estimate hazard ratios (HRs) for outcomes of interest.

**Results:**

The GLORIA-AF Phase III registry enrolled 21,300 patients between January 2014 and December 2016. Of these, 3839 were prescribed dabigatran, 4015 rivaroxaban and 4505 apixaban, with median ages of 71.0, 71.0, and 73.0 years, respectively. In the PS-matched set, the adjusted HRs and 95% confidence intervals (CIs) for dabigatran vs rivaroxaban were, for stroke: 1.27 (0.79–2.03), major bleeding 0.59 (0.40–0.88), myocardial infarction 0.68 (0.40–1.16), and all-cause death 0.86 (0.67–1.10). For the comparison of dabigatran vs apixaban, in the PS-matched set, the adjusted HRs were, for stroke 1.16 (0.76–1.78), myocardial infarction 0.84 (0.48–1.46), major bleeding 0.98 (0.63–1.52) and all-cause death 1.01 (0.79–1.29). For the comparison of rivaroxaban vs apixaban, in the PS-matched set, the adjusted HRs were, for stroke 0.78 (0.52–1.19), myocardial infarction 0.96 (0.63–1.45), major bleeding 1.54 (1.14–2.08), and all-cause death 0.97 (0.80–1.19).

**Conclusions:**

Patients treated with dabigatran had a 41% lower risk of major bleeding compared with rivaroxaban, but similar risks of stroke, MI, and death. Relative to apixaban, patients treated with dabigatran had similar risks of stroke, major bleeding, MI, and death. Rivaroxaban relative to apixaban had increased risk for major bleeding, but similar risks for stroke, MI, and death.

**Registration:**

URL: https://www.clinicaltrials.gov. Unique identifiers: NCT01468701, NCT01671007. Date of registration: September 2013.

**Graphical abstract:**

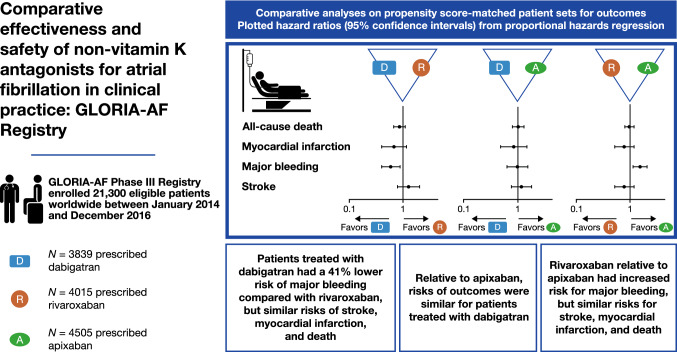

**Supplementary Information:**

The online version contains supplementary material available at 10.1007/s00392-022-01996-2.

## Introduction

The non-vitamin K antagonists (NOACs) have changed the landscape of stroke prevention in patients with atrial fibrillation (AF) [1, 2]. In randomized clinical trials (RCTs) and large observational studies, NOACs showed favorable benefit-risk profiles compared with vitamin K antagonists (VKAs) [3–8]. Hence, current clinical practice guidelines recommend NOACs for prevention of ischemic stroke in patients with AF [9–12].

Currently, four NOACs are available for clinical use in patients with AF, including the direct thrombin inhibitor dabigatran and the factor Xa inhibitors rivaroxaban, apixaban, and edoxaban [3–6], but RCTs directly comparing these agents are lacking and available comparisons of individual NOACs are retrospective [13–17]. Studies based on claims databases have limitations in terms of data quality and follow-up duration. Prospective registries and cohort studies can provide more complete and accurate data, longer follow-up [18], and capture variables not included in claims databases [18, 19].

The Global Registry on Long-Term Oral Antithrombotic Treatment in Patients with Atrial Fibrillation (GLORIA-AF) was a large, global, prospective registry providing comparative data on the use of NOACs in clinical practice. For this report, we performed a head-to-head comparison of NOACs for the outcomes of interest using the Phase III, final 3-year follow-up period of the GLORIA-AF Registry.

## Methods

### Study design and setting

The 3-phase design of the GLORIA-AF Registry Program has been described [20]. Consecutive patients ≥ 18 years old with recently identified AF and CHA_2_DS_2_-VASc scores ≥ 1, meeting the inclusion criteria (Online Resource: Methods 1) were enrolled and managed according to local clinical practice at the discretion of treating physicians. Patients in Phase III were followed for 3 years, regardless of antithrombotic therapy. The study was governed by Good Clinical Practice and the Declaration of Helsinki. The protocol was approved by the European Medicines Agency and institutional review boards at each participating site. Patients provided written informed consent. An independent academic steering committee oversaw the design, execution, study conduct, and manuscript development (Online Resource: Data Sharing Statement).

### Clinical outcomes

The outcomes of interest were stroke (hemorrhagic, ischemic, and uncertain classification), major bleeding (International Society on Thrombosis and Haemostasis criteria), myocardial infarction, and all-cause death. Furthermore, the composite outcome of stroke, systemic embolism, myocardial infarction, vascular death, and life-threatening bleeding events was also analyzed (life-threatening bleeding events defined in Methods 2; Online Resource).

### Statistical methods

Demographics and baseline characteristics were summarized descriptively and compared for dabigatran vs rivaroxaban, dabigatran vs apixaban, and rivaroxaban vs apixaban within different patient sets using standardized differences. Categorical variables were summarized by frequencies and percentages, and continuous variables as means and standard deviations (SD). For analyses comparing dabigatran vs rivaroxaban, dabigatran vs apixaban, and rivaroxaban vs apixaban, missing data for baseline covariates and cause of death were handled using multiple imputation (Online Resource: Methods 3). Descriptive analyses for the propensity score (PS)-trimmed and PS-matched sets are based on the PS calculated using the first of the multiple imputation patient sets, i.e., the first trimmed and matched sets. Outcome analyses were performed separately for each imputed patient set, and results were combined to provide estimates under the missing-at-random assumption. The PS was calculated separately for dabigatran vs rivaroxaban, dabigatran vs apixaban, and rivaroxaban vs apixaban, following which restricted sets and matched sets for the pairs compared were then derived. For each of the pairwise comparisons, outcome analyses were performed separately for the patient sets described below. Data were analyzed using SAS® software version 9.4 or later (SAS Institute, Inc., Cary, NC).

### Patient sets

*Post hoc* comparisons of dabigatran vs rivaroxaban, dabigatran vs apixaban, and rivaroxaban vs apixaban were performed following the same methodology as defined for the analyses of the relative effectiveness and safety of dabigatran vs VKA among the PS-trimmed and PS-matched patient sets. The PS-trimmed set consisted of the cohort obtained after excluding those in the nonoverlapping tails of the PS distribution (PS-trimming) within each geographical region (Online Resource: Fig. 1, Fig. 2, and Fig. 3). Excluding patients from the tails of the PS distribution addresses channeling bias and improves the validity of comparisons. The PS-matched sets were generated from the PS-trimmed patient sets by 1:1 greedy nearest-neighbor matching of patients on dabigatran to those on rivaroxaban, dabigatran to apixaban, and rivaroxaban to apixaban, with a predefined caliper within the region (Online Resource: Methods 4). Descriptive analyses for the PS-trimmed and PS-matched sets were based on the PS calculated using the first trimmed and matched sets.

### Clinical outcome analyses

Incidence rates with 95% confidence intervals (CIs) for key outcome events were calculated for dabigatran vs rivaroxaban, dabigatran vs apixaban, and rivaroxaban vs apixaban within the trimmed and matched patient sets. The initial analysis comparing effects of NOACs was conducted using a multivariable Cox regression model within the PS-trimmed patient set. The model included core variables (e.g., treatment, age, sex, and risk factors for stroke and bleeding). Further variables were included based on covariate selection procedures (Methods 5; Online Resource). Hazard ratios (HRs) with 95% CIs were presented for outcomes considered. The comparative analyses for dabigatran vs rivaroxaban, dabigatran vs apixaban, and rivaroxaban vs apixaban were also conducted in the PS-matched patient set by Cox regression with a shared frailty factor to adjust matching [21]. Among matched patients, the balance between the treatment groups was compared for individual, prespecified covariates (Online Resource: Table 1), and covariates with a standardized difference > 10% were considered unbalanced and included as a separate variable in the final regression model. Kaplan–Meier curves were plotted based on the matched patients for graphical comparison. Additionally, we conducted a PS stratification analysis, based on strata formed by deciles of an extended PS and geographic region (Online Resource: Methods 6). Longitudinal outcomes were analyzed on an as-treated basis, censoring patients after permanent discontinuation of initial treatment or study termination.

## Results

The GLORIA-AF Phase III registry included 21,591 patients enrolled at 935 sites in 38 countries, of whom 21,300 were eligible for analysis. Approximately 48% were enrolled in Europe, 24% in North America, 20% in Asia, and 8% in Latin America. The eligible patient population included 19,718 patients who received at least 1 dose of prescribed antithrombotic treatment and 1142 who did not initiate the prescribed antithrombotic treatment at baseline. Of the treated patients, 12,577 (60.3%) received a NOAC, either dabigatran (*n* = 3839), rivaroxaban (*n* = 4015), apixaban (*n* = 4505), or edoxaban (*n* = 332). Because of the small number of patients taking edoxaban, it was not further assessed. Baseline characteristics of patients treated with dabigatran, rivaroxaban, and apixaban are provided in the Online Resource (Table 2**)**. A total of 17,140 (80.5%) patients completed the full 3 years of observation. When possible, information on vital status was collected for patients who did not complete the planned observation period, and at the end of the study was available for all but 997 (4.7%) patients.

### Comparisons of dabigatran vs rivaroxaban

#### PS-trimmed cohorts

The PS-trimmed set included 3618 patients treated with dabigatran and 3785 treated with rivaroxaban (Table 1A). The majority came from Europe (54.0% in the dabigatran group and 50.4% in the rivaroxaban group), but the proportion of patients prescribed dabigatran was higher in Asia (24.0% dabigatran vs 9.2% rivaroxaban) and Latin America (10.8% dabigatran vs 7.1% rivaroxaban); while in North America, rivaroxaban was prescribed more often (33.4% rivaroxaban vs 11.1% dabigatran). In terms of other characteristics, the dabigatran and rivaroxaban populations were similar, the PS density plots showing considerable overlap (Online Resource: Fig. 1). Patients treated with rivaroxaban more often had paroxysmal AF (57.4% rivaroxaban vs 54.1% dabigatran), coronary artery disease (16.5 vs 12.8%), and diabetes mellitus (24.5 vs 21.5%), but less often had previous stroke (9.6 vs 7.1%) than dabigatran-treated patients. Concomitant antiplatelet therapy was more frequent in patients given rivaroxaban (18.3 vs 12.6%). Half the dabigatran-treated patients took 150 mg twice daily [BID] and 44.9% took 110 mg BID; over 75% of patients treated with rivaroxaban received 20 mg daily [OD].

**Table 1:** Baseline characteristics of dabigatran- and rivaroxaban-treated patients within the PS-trimmed cohort (A) and the PS-matched cohort (B)

Characteristics	(A) PS-trimmed cohort			(B) PS-matched cohort		
	Dabigatran *N* = 3618	Rivaroxaban *N* = 3785	Standardized difference*	Dabigatran *N* = 2918	Rivaroxaban *N* = 2918	Standardized difference*
Age, years, *n* (%)						
< 65	903 (25.0)	951 (25.1)	− 0.0039	667 (22.9)	693 (23.7)	− 0.0211
65–74	1450 (40.1)	1442 (38.1)	0.0406	1172 (40.2)	1110 (38.0)	0.0436
≥ 75	1265 (35.0)	1392 (36.8)	− 0.0378	1079 (37.0)	1115 (38.2)	− 0.0255
Female sex, *n* (%)	1644 (45.4)	1685 (44.5)	0.0185	1348 (46.2)	1296 (44.4)	0.0358
Creatinine clearance, mL/min, *n* (%)						
< 30	37 (1.0)	52 (1.4)	− 0.0323	25 (0.9)	43 (1.5)	− 0.0575
30 to < 50	305 (8.4)	377 (10.0)	− 0.0530	248 (8.5)	311 (10.7)	− 0.0734
50 to < 80	1201 (33.2)	1182 (31.2)	0.0421	964 (33.0)	966 (33.1)	− 0.0015
≥ 80	1242 (34.3)	1527 (40.3)	− 0.1246	1047 (35.9)	1085 (37.2)	− 0.0270
Missing	833 (23.0)	647 (17.1)		634 (21.7)	513 (17.6)	
Type of AF, *n* (%)						
Paroxysmal	1956 (54.1)	2171 (57.4)	− 0.0664	1553 (53.2)	1580 (54.1)	− 0.0186
Persistent	1232 (34.1)	1302 (34.4)	− 0.0073	997 (34.2)	1048 (35.9)	− 0.0366
Permanent	430 (11.9)	312 (8.2)	0.1213	368 (12.6)	290 (9.9)	0.0846
Medical history, *n* (%)						
Congestive heart failure	649 (17.9)	655 (17.3)	0.0166	567 (19.4)	547 (18.7)	0.0174
History of hypertension	2713 (75.0)	2870 (75.8)	− 0.0195	2222 (76.1)	2182 (74.8)	0.0319
Diabetes mellitus	777 (21.5)	926 (24.5)	− 0.0711	672 (23.0)	691 (23.7)	− 0.0154
Previous stroke	348 (9.6)	268 (7.1)	0.0918	289 (9.9)	211 (7.2)	0.0956
Coronary artery disease	462 (12.8)	626 (16.5)	− 0.1067	390 (13.4)	447 (15.3)	− 0.0558
Prior bleeding	113 (3.1)	164 (4.3)	− 0.0639	101 (3.5)	120 (4.1)	− 0.0341
Alcohol abuse (> 8 units/week)	208 (5.7)	280 (7.4)	− 0.0666	199 (6.8)	204 (7.0)	− 0.0068
Current smoker	348 (9.6)	299 (7.9)	0.0608	268 ( 9.2)	246 (8.4)	0.0266
Past smoker	874 (24.2)	1264 (33.4)	− 0.2051	767 (26.3)	875 (30.0)	− 0.0824
Previous OAC use within 3 months	1594 (44.1)	2060 (54.4)	− 0.2085	1275 (43.7)	1439 (49.3)	− 0.1129
Chronic concomitant medications, *n* (%)						
Antiplatelet	457 (12.6)	694 (18.3)	− 0.1582	407 (13.9)	441 (15.1)	− 0.0331
Drugs with higher bleeding risk (HAS-BLED)#	515 (14.2)	806 (21.3)	− 0.1855	460 (15.8)	496 (17.0)	− 0.0333
Region, *n* (%)						
Asia	869 (24.0)	347 (9.2)	0.4074	347 (11.9)	347 (11.9)	0.0000
Europe	1955 (54.0)	1906 (50.4)	0.0737	1903 (65.2)	1903 (65.2)	0.0000
North America	403 (11.1)	1264 (33.4)	− 0.5552	400 (13.7)	400 (13.7)	0.0000
Latin America	391 (10.8)	268 (7.1)	0.1309	268 (9.2)	268 (9.2)	0.0000
Treatment dose, *n* (%)						
	150 mg BID: 1915 (52.9)	10 mg OD: 101 (2.7)	–	150 mg BID: 1698 (58.2)	10 mg OD: 98 (3.4)	–
	110 mg BID: 1624 (44.9)	15 mg OD: 792 (20.9)	–	110 mg BID: 1151 (39.4)	15 mg OD: 674 (23.1)	–
	75 mg BID: 50 (1.4)	20 mg OD: 2867 (75.7)	–	75 mg BID: 47 (1.6)	20 mg OD: 2121 (72.7)	–
	Other dose: 29 (0.8)	Other dose: 25 (0.7)	–	Other dose: 22 (0.8)	Other dose: 25 (0.9)	–

*PS* propensity score; *AF* atrial fibrillation; *HAS-BLED* hypertension, abnormal liver/renal function, stroke history, bleeding history or predisposition, labile international normalized ratio (INR), elderly (age > 65 years), drug/alcohol usage; *OAC* oral anticoagulant; *OD* once daily; *BID* twice daily; *mg* milligram

*Standardized difference > 10% (in absolute value) is considered unbalanced between the two treatment groups

# Concomitant use of drugs associated with higher bleeding risk (i.e., antiplatelet agent, Cox-2 inhibitor, or other non-steroidal, anti-inflammatory drug)

The incidence rates for outcomes of interest within the PS-trimmed patient set are shown in Table 2A for patients treated with dabigatran and rivaroxaban. Cox regression analysis within this patient set found that patients treated with dabigatran had a lower rate of major bleeding (HR: 0.58; 95% CI: 0.41–0.82; Table 3). Risks of stroke (HR: 1.40; 95% CI: 0.94–2.09), myocardial infarction (HR: 0.69; 95% CI: 0.44–1.09), all-cause death (HR: 0.85; 95% CI: 0.69–1.05), and the composite outcome (HR: 0.90; 95% CI: 0.73–1.11) were similar with these anticoagulants.

**Table 2:** Incidence rates for dabigatran- and rivaroxaban-treated patients within the PS-trimmed cohort (A) and the PS-matched cohort (B)

Incidence rates/100 patient-years (95% CI)	(A) PS-trimmed cohort		(B) PS-matched cohort	
	Dabigatran *n* = 3628	Rivaroxaban *n* = 3782	Dabigatran *n* = 2896	Rivaroxaban *n* = 2896
Composite outcome*	2.21 (1.88–2.55)	2.52 (2.17–2.87)	2.24 (1.85–2.66)	2.37 (1.96–2.80)
Stroke	0.78 (0.59–0.99)	0.54 (0.38–0.71)	0.71 (0.50–0.95)	0.54 (0.36–0.73)
GI bleeding	0.34 (0.21–0.48)	0.77 (0.59–0.96)	0.37 (0.23–0.55)	0.55 (0.36–0.75)
ICH bleeding	0.17 (0.09–0.28)	0.25 (0.15–0.36)	0.16 (0.06–0.27)	0.24 (0.12–0.37)
Major bleeding	0.67 (0.49–0.86)	1.47 (1.21–1.72)	0.68 (0.48–0.90)	1.15 (0.89–1.43)
Myocardial infarction	0.41 (0.28–0.56)	0.66 (0.49–0.85)	0.42 (0.26–0.60)	0.60 (0.41–0.83)
All-cause death	2.06 (1.75–2.38)	2.49 (2.16–2.84)	2.17 (1.80–2.57)	2.45 (2.05–2.85)

*PS *propensity score; *CI* confidence interval; *GI* gastrointestinal; *ICH* intracerebral

^*^Composite outcome of stroke-systemic embolism, myocardial infarction, life-threatening bleeding events, and vascular death

**Table 3:** Cox regression analysis for dabigatran vs rivaroxaban in the PS-trimmed and PS-matched cohorts and* post hoc* PS extended sensitivity analysis

Hazard ratios (95% CI)	PS-matched cohort*	PS-trimmed cohort	*Post hoc* PS stratification with extended PS
	Dabigatran (*n* = 2896) Rivaroxaban (*n* = 2896)	Dabigatran (*n* = 3628) Rivaroxaban (*n* = 3782)	Dabigatran (*n* = 3605) Rivaroxaban (*n* = 3714)
Composite outcome	0.93 (0.73–1.19)	0.90 (0.73–1.11)	0.97 (0.77–1.21)
Stroke	1.27 (0.79–2.03)	1.40 (0.94–2.09)	1.53 (1.00–2.33)
Major bleeding	0.59 (0.40–0.88)	0.58 (0.41–0.82)	0.58 (0.40–0.85)
Myocardial infarction	0.68 (0.40–1.16)	0.69 (0.44–1.09)	0.70 (0.43–1.12)
All-cause death	0.86 (0.67–1.10)	0.85 (0.69–1.05)	0.90 (0.72–1.13)

Variables that were selected for primary analysis and PS adjustment analysis can be found in the Supplemental material

*PS* propensity score; *CI* confidence interval

^*^Unbalanced covariates: History of previous stroke, transient ischemic attack, or systemic embolism; previous oral anticoagulant use within 3 months

#### PS-matched cohorts

The PS-matched set consisted of 2918 patients in each of the two treated groups, whose baseline characteristics are shown in Table 1B. Most were enrolled in Europe (65.2%). Of the dabigatran group, 58.2% of patients were prescribed 150 mg BID, and 39.4% received 110 mg BID; of the rivaroxaban group, 72.7% of patients received 20 mg OD. The incidence rate for the key outcomes within the PS-matched cohort are shown in Table 2B.

Cox regression analysis of the PS-matched patient set, adjusted for unbalanced variables, revealed that treatment with dabigatran was associated with a lower rate of major bleeding (HR: 0.59; 95% CI: 0.40–0.88; Table 3). Rates of stroke (HR: 1.27; 95% CI: 0.79–2.03), MI (HR: 0.68; 95% CI: 0.40–1.16), all-cause death (HR: 0.86; 95% CI: 0.67–1.10), and the composite outcome (HR: 0.93; 95% CI: 0.73–1.19) were similar for dabigatran and rivaroxaban. *Post hoc* sensitivity analyses using an extended set of covariates in the propensity score confirmed the PS-matched analysis (Table 3).

### Comparisons of dabigatran vs apixaban

#### PS-trimmed cohorts

The PS-trimmed set included 3580 patients treated with dabigatran and 4154 patients treated with apixaban; their baseline characteristics are shown in Table 4A. Nearly half (49.2%) of patients treated with apixaban were enrolled in Europe, and 39% were in North America. Apixaban-treated patients had more comorbid conditions, including hypertension (77.1% apixaban vs 74.7% dabigatran), diabetes mellitus (22.7 vs 21.0%), coronary artery disease (18.2 vs 12.8%), chronic kidney disease (creatinine clearance < 50 mL/min: 14.2 vs 9.5%), and prior bleeding (4.4 vs 3.2%) compared with the dabigatran group. Concomitant antiplatelet therapy was more common among apixaban-treated patients (19.7 vs 12.8%). These differences are reflected in the PS distributions for patients initiating dabigatran and apixaban (Online Resource: Fig. 2).

**Table 4:** Baseline characteristics of dabigatran- and apixaban-treated patients within the PS-trimmed cohort (A) and the PS-matched cohort (B)

Characteristics	(A) PS-trimmed cohort			(B) PS-matched cohort		
	Dabigatran *N* = 3580	Apixaban *N* = 4154	Standardized difference*	Dabigatran *N* = 2694	Apixaban *N* = 2694	Standardized difference*
Age, years, *n* (%)						
< 65	855 (23.9)	832 (20.0)	0.0932	609 (22.6)	527 (19.6)	0.0747
65–74	1450 (40.5)	1505 (36.2)	0.0879	1018 (37.8)	1041 (38.6)	− 0.0176
≥ 75	1275 (35.6)	1817 (43.7)	− 0.1667	1067 (39.6)	1126 (41.8)	− 0.0446
Female sex, *n* (%)	1644 (45.9)	1960 (47.2)	− 0.0253	1153 (42.8)	1253 (46.5)	− 0.0747
Creatinine clearance, mL/min, *n* (%)						
< 30	38 (1.1)	67 (1.6)	− 0.0480	29 (1.1)	42 (1.6)	− 0.0423
30 to < 50	303 (8.5)	524 (12.6)	− 0.1355	234 (8.7)	351 (13.0)	− 0.1399
50 to < 80	1206 (33.7)	1358 (32.7)	0.0211	921 (34.2)	899 (33.4)	0.0173
≥ 80	1212 (33.9)	1499 (36.1)	− 0.0468	948 (35.2)	958 (35.6)	− 0.0078
Missing	821 (22.9)	706 (17.0)		562 (20.9)	444 (16.5)	
Type of AF, *n* (%)						
Paroxysmal	1950 (54.5)	2409 (58.0)	− 0.0711	1473 (54.7)	1419 (52.7)	0.0402
Persistent	1212 (33.9)	1423 (34.3)	− 0.0085	904 (33.6)	1011 (37.5)	− 0.0831
Permanent	418 (11.7)	322 (7.8)	0.1328	317 (11.8)	264 (9.8)	0.0635
Medical history, *n* (%)						
Congestive heart failure	620 (17.3)	690 (16.6)	0.0189	480 (17.8)	454 (16.9)	0.0255
History of hypertension	2676 (74.7)	3203 (77.1)	− 0.0552	2012 (74.7)	2033 (75.5)	− 0.0180
Diabetes mellitus	753 (21.0)	944 (22.7)	− 0.0409	620 (23.0)	536 (19.9)	0.0760
Previous stroke	408 (11.4)	481 (11.6)	− 0.0057	335 (12.4)	331 (12.3)	0.0045
Coronary artery disease	458 (12.8)	757 (18.2)	− 0.1504	401 (14.9)	384 (14.3)	0.0179
Prior bleeding	115 (3.2)	184 (4.4)	− 0.0635	100 (3.7)	103 (3.8)	− 0.0058
Alcohol abuse (> 8 units/week)	211 (5.9)	325 (7.8)	− 0.0764	199 (7.4)	202 (7.5)	− 0.0042
Current smoker	351 (9.8)	349 (8.4)	0.0488	292 (10.8)	259 (9.6)	0.0404
Past smoker	868 (24.2)	1423 (34.3)	− 0.2214	763 (28.3)	779 (28.9)	− 0.0131
Previous OAC use within 3 months	1576 (44.0)	2033 (48.9)	− 0.0987	1142 (42.4)	1165 (43.2)	− 0.0173
Chronic concomitant medications, *n* (%)						
Antiplatelet	459 (12.8)	818 (19.7)	− 0.1870	404 (15.0)	400 (14.8)	0.0042
Drugs with higher bleeding risk (HAS-BLED)#	519 (14.5)	952 (22.9)	− 0.2172	458 (17.0)	478 (17.7)	− 0.0196
Region, *n* (%)						
Asia	836 (23.4)	338 (8.1)	0.4272	336 (12.5)	336 (12.5)	0.0000
Europe	1972 (55.1)	2045 (49.2)	0.1174	1811 (67.2)	1811 (67.2)	0.0000
North America	401 (11.2)	1621 (39.0)	− 0.6774	400 (14.8)	400 (14.8)	0.0000
Latin America	371 (10.4)	150 (3.6)	0.2672	147 (5.5)	147 (5.5)	0.0000
Treatment dose, *n* % (%)						
	150 mg BID: 1882 (52.6)	5 mg BID: 3374 (81.2)	–	150 mg BID: 1553 (57.6)	5 mg BID: 2145 (79.6)	–
	110 mg BID: 1618 (45.2)	2.5 mg BID: 769 (18.5)	–	110 mg BID: 1079 (40.1)	2.5 mg BID: 544 (20.2)	–
	75 mg BID: 51 (1.4)	Other dose: 11 (0.3)	–	75 mg BID: 43 (1.6)	Other dose: 5 (0.2)	–
	Other dose: 29 (0.8)		–	Other dose: 19 (0.7)		–

*PS* propensity score; *AF* atrial fibrillation; *HAS-BLED* hypertension, abnormal liver/renal function, stroke history, bleeding history or predisposition, labile international normalized ratio (INR), elderly (age > 65 years), drug/alcohol usage; *OAC* oral anticoagulant; *BID* twice daily; *mg* milligram

^*^ Standardized difference > 10% (in absolute value) is considered unbalanced between the two treatment groups

^#^ Concomitant use of drugs associated with higher bleeding risk (i.e., antiplatelet agent, Cox-2 inhibitor, or other non-steroidal, anti-inflammatory drug)

Half of dabigatran-treated patients received 150 mg BID and 45.2% received 110 mg BID; over 80% of patients treated with apixaban received 5 mg BID. Incidence rates for the outcomes of interest in patients treated with dabigatran vs apixaban are shown in Table 5A.

**Table 5:** Incidence rates for dabigatran- and apixaban-treated patients within the PS-trimmed cohort (A) and the PS-matched cohort (B)

Incidence rates/100 patient-years (95% CI)	(A) PS-trimmed cohort		(B) PS-matched cohort	
	Dabigatran *n* = 3585	Apixaban *n* = 4145	Dabigatran *n* = 2683	Apixaban *n* = 2683
Composite outcome*	2.35 (2.00–2.72)	2.55 (2.22–2.90)	2.55 (2.12–2.98)	2.30 (1.90–2.69)
Stroke	0.85 (0.65–1.07)	0.73 (0.56–0.90)	0.86 (0.62–1.13)	0.77 (0.54–1.02)
GI bleeding	0.35 (0.22–0.50)	0.31 (0.20–0.42)	0.41 (0.25–0.58)	0.21 (0.10–0.35)
ICH bleeding	0.18 (0.08–0.28)	0.24 (0.15–0.35)	0.17 (0.07–0.28)	0.22 (0.11–0.33)
Major bleeding	0.68 (0.50–0.86)	0.93 (0.74–1.12)	0.75 (0.53–0.98)	0.78 (0.56–1.02)
Myocardial infarction	0.42 (0.28–0.57)	0.71 (0.55–0.89)	0.45 (0.28–0.63)	0.55 (0.37–0.75)
All-cause death	2.18 (1.84–2.51)	2.71 (2.39–3.05)	2.35 (1.96–2.74)	2.56 (2.16–2.98)

*PS* propensity score; *CI* confidence interval; *GI* gastrointestinal; *ICH* intracerebral

^*^Composite outcome of stroke, systemic embolism, myocardial infarction, life-threatening bleeding events, and vascular death

#### PS-matched cohorts

After PS-matching, the study consisted of 2694 patients in each group. In the apixaban group, 79.6% of patients were treated with 5 mg BID, while only 57.6% of patients in the dabigatran group received 150 mg BID. Baseline characteristics are shown in Table 4B, and incidence rates for the outcomes of interest in Table 5B.

Cox regression analysis of the PS-matched patient set, adjusted for the unbalanced variables, revealed similar risks for stroke (HR: 1.16; 95% CI: 0.76–1.78), major bleeding (HR: 0.98; 95% CI: 0.63–1.52), myocardial infarction (HR: 0.84; 95% CI: 0.48–1.46), all-cause death (HR: 1.01; 95% CI: 0.79–1.29), and the composite outcome (HR: 1.17; 95% CI: 0.91–1.51), with dabigatran relative to apixaban (Table 6). An extended PS stratified sensitivity analysis adjusted for unbalanced variables confirmed the findings of the PS-matched analysis.

**Table 6:** Cox regression analysis for dabigatran vs apixaban in the PS-trimmed and PS-matched cohorts and* post hoc* PS extended sensitivity analysis

Hazard ratios (95% CI)	PS-matched cohort*	PS-trimmed cohort	*Post hoc* PS stratification with extended PS
	Dabigatran (*n* = 2683) Apixaban (*n* = 2683)	Dabigatran (*n* = 3585) Apixaban (*n* = 4145)	Dabigatran (*n* = 3565) Apixaban (*n* = 4101)
Composite outcome	1.17 (0.91–1.51)	1.10 (0.88–1.37)	1.14 (0.92–1.43)
Stroke	1.16 (0.76–1.78)	1.26 (0.89–1.77)	1.19 (0.83–1.69)
Major bleeding	0.98 (0.63–1.52)	0.93 (0.64–1.36)	0.98 (0.67–1.43)
Myocardial infarction	0.84 (0.48–1.46)	0.71 (0.46–1.10)	0.72 (0.45–1.17)
All-cause death	1.01 (0.79–1.29)	0.90 (0.74–1.10)	0.95 (0.77–1.18)

Variables that were selected for primary analysis and PS adjustment analysis can be found in the Supplemental material

*PS* propensity score; *CI* confidence interval

^*^Unbalanced covariates: creatinine clearance

### Comparisons of rivaroxaban vs apixaban

#### PS-trimmed cohorts

The PS-trimmed set included 3789 patients treated with rivaroxaban and 4227 treated with apixaban (Table 7A). The majority of patients came from Europe (50.1% in the rivaroxaban group and 49.2% in the apixaban group), but the proportion of patients prescribed apixaban was higher in North America (38.8% apixaban vs 33.7% rivaroxaban), while in Latin America, the proportion of patients receiving rivaroxaban was higher (7.1% rivaroxaban vs 3.6% apixaban). In terms of other characteristics, the rivaroxaban and apixaban populations were similar and the PS density plots showed considerable overlap (Online Resource: Fig. 3). Notably, the proportion of patients over age 75 years more often received apixaban compared with rivaroxaban (44.1% apixaban vs 37.4% rivaroxaban). Patients treated with apixaban more often had previous history of stroke, transient ischemic attack, or systemic embolism (16.3% apixaban vs 10.8% rivaroxaban). Previous use of an oral anticoagulant within 3 months prior to the baseline visit was more prevalent in rivaroxaban patients (54.7%) compared with apixaban patients (49.0%). Among rivaroxaban-treated patients, 75.4% received 20 mg OD and the corresponding proportion of apixaban patients receiving a standard dose (5 mg BID) was 80.9%.

**Table 7:** Baseline characteristics of rivaroxaban- and apixaban-treated patients within the PS-trimmed cohort (A) and the PS-matched cohort (B)

Characteristics	(A) PS-trimmed cohort			(B) PS-matched cohort		
	Rivaroxaban *N* = 3789	Apixaban *N* = 4227	Standardized difference*	Rivaroxaban *N* = 3559	Apixaban *N* = 3559	Standardized difference*
Age, years, *n* (%)						
< 65	905 (23.9)	829 (19.6)	− 0.1037	844 (23.7)	790 (22.2)	− 0.0361
65–74	1466 (38.7)	1533 (36.3)	− 0.0501	1373 (38.6)	1349 (37.9)	− 0.0139
≥ 75	1418 (37.4)	1865 (44.1)	0.1366	1342 (37.7)	1420 (39.9)	0.0450
Female sex, *n* (%)	1704 (45.0)	1984 (46.9)	0.0394	1590 (44.7)	1616 (45.4)	0.0147
Creatinine clearance, mL/min, *n* (%)						
< 30	55 (1.5)	69 (1.6)	0.0147	49 (1.4)	54 (1.5)	0.0118
30 to < 50	372 (9.8)	549 (13.0)	0.0999	348 (9.8)	414 (11.6)	0.0600
50 to < 80	1204 (31.8)	1372 (32.5)	0.0146	1140 (32.0)	1122 (31.5)	− 0.0109
≥ 80	1506 (39.7)	1517 (35.9)	− 0.0796	1433 (40.3)	1359 (38.2)	− 0.0426
Missing	652 (17.2)	720 (17.0)	− 0.0046	589 (16.5)	610 (17.1)	0.0158
Type of AF, *n* (%)						
Paroxysmal	2168 (57.2)	2460 (58.2)	0.0198	2038 (57.3)	2021 (56.8)	− 0.0096
Persistent	1312 (34.6)	1428 (33.8)	− 0.0178	1240 (34.8)	1247 (35.0)	0.0041
Permanent	309 (8.2)	339 (8.0)	−0.0050	281 (7.9)	291 (8.2)	0.0103
Medical history, *n* (%)						
Congestive heart failure	665 (17.6)	727 (17.2)	− 0.0093	613 (17.2)	627 (17.6)	0.0104
History of hypertension	2858 (75.4)	3259 (77.1)	0.0393	2684 (75.4)	2696 (75.8)	0.0078
Diabetes mellitus	883 (23.3)	965 (22.8)	− 0.0113	829 (23.3)	810 (22.8)	− 0.0127
Previous stroke	260 (6.9)	472 (11.2)	0.1507	255 (7.2)	246 (6.9)	− 0.0099
Coronary artery disease	639 (16.9)	785 (18.6)	0.0447	620 (17.4)	631 (17.7)	0.0081
Prior bleeding	189 (5.0)	225 (5.3)	0.0151	182 (5.1)	182 (5.1)	0.0000
Alcohol abuse (> 8 units/week)	297 (7.8)	351 (8.3)	0.0171	293 (8.2)	298 (8.4)	0.0051
Current smoker	306 (8.1)	344 (8.1)	0.0023	295 (8.3)	292 (8.2)	− 0.0031
Past smoker	1297 (34.2)	1472 (34.8)	0.0125	1245 (35.0)	1261 (35.4)	0.0094
Previous OAC use within 3 months	2072 (54.7)	2072 (49.0)	− 0.1136	1944 (54.6)	1718 (48.3)	− 0.1273
Chronic concomitant medications, *n* (%)						
Antiplatelet	705 (18.6)	837 (19.8)	0.0303	685 (19.2)	705 (19.8)	0.0142
Drugs with higher bleeding risk (HAS-BLED)#	820 (21.6)	972 (23.0)	0.0325	798 (22.4)	818 (23.0)	0.0134
Region, *n* (%)						
Asia	344 (9.1)	360 (8.5)	−0.0198	290 (8.1)	290 (8.1)	0.0000
Europe	1900 (50.1)	2078 (49.2)	−0.0197	1846 (51.9)	1846 (51.9)	0.0000
North America	1277 (33.7)	1638 (38.8)	0.1052	1274 (35.8)	1274 (35.8)	0.0000
Latin America	268 (7.1)	151 (3.6)	−0.1564	149 (4.2)	149 (4.2)	0.0000
Treatment dose, *n* (%)						
	10 mg OD: 100 (2.6)	5 mg BID: 3420 (80.9)	–	10 mg OD: 89 (2.5)	5 mg BID: 2911 (81.8)	–
	15 mg OD: 806 (21.3)	2.5 mg BID: 796 (18.8)	–	15 mg OD: 735 (20.7)	2.5 mg BID: 640 (18.0)	–
	20 mg OD: 2856 (75.4)	Other dose: 11 (0.3)	–	20 mg OD: 2711 (76.2)	Other dose: 8 (0.2)	–
	Other dose: 27 (0.7)		–	Other dose: 24 (0.7)		–

*PS* propensity score; *AF* atrial fibrillation; *HAS-BLED* hypertension, abnormal liver/renal function, stroke history, bleeding history or predisposition, labile international normalized ratio (INR), elderly (age > 65 years), drug/alcohol usage; *OAC* oral anticoagulant; *OD* once daily; *BID* twice daily; *mg* milligram

^*^ Standardized difference > 10% (in absolute value) is considered unbalanced between the two treatment groups

^#^ Concomitant use of drugs associated with higher bleeding risk (i.e., antiplatelet agent, Cox-2 inhibitor, or other non-steroidal anti-inflammatory drug)

The incidence rates for outcomes of interest within the PS-trimmed patient set are shown in Table 8A for patients treated with rivaroxaban and apixaban. Cox regression analysis within this patient set found that patients treated with rivaroxaban had a higher rate of major bleeding (HR: 1.61; 95% CI: 1.22–2.12; Table 9). Risks of stroke (HR: 0.83; 95% CI: 0.57–1.21), myocardial infarction (HR: 0.97; 95% CI: 0.67–1.39), all-cause death (HR: 1.06; 95% CI: 0.89–1.28), and the composite outcome (HR: 1.04; 95% CI: 0.86–1.26) were similar with these anticoagulants.

**Table 8:** Incidence rates for rivaroxaban- and apixaban-treated patients within the PS-trimmed cohort (A) and the PS-matched cohort (B)

Incidence rates/100 patient-years (95% CI)	(A) PS-trimmed cohort		(B) PS-matched cohort	
	Rivaroxaban *n* = 3792	Apixaban *n* = 4223	Rivaroxaban *n* = 3563	Apixaban *n* = 3563
Composite outcome*	2.57 (2.23–2.94)	2.62 (2.28–2.96)	2.56 (2.19–2.94)	2.49 (2.11–2.85)
Stroke	0.57 (0.41–0.74)	0.75 (0.58–0.93)	0.56 (0.39–0.74)	0.70 (0.51–0.90)
Major bleeding	1.44 (1.19–1.71)	0.93 (0.75–1.13)	1.48 (1.22–1.76)	0.95 (0.74–1.17)
Myocardial infarction	0.65 (0.49–0.83)	0.72 (0.56–0.89)	0.65 (0.47–0.84)	0.67 (0.49–0.85)
All-cause death	2.63 (2.29–2.98)	2.75 (2.43–3.08)	2.59 (2.24–2.96)	2.68 (2.33–3.03)

*PS* propensity score; *CI* confidence interval

^*^ Composite outcome of stroke, systemic embolism, myocardial infarction, life-threatening bleeding events, and vascular death

**Table 9:** Cox regression analysis for rivaroxaban and apixaban in the PS-trimmed and PS-matched cohorts and *post hoc* PS extended sensitivity analysis

Hazard ratios (95% CI)	PS-matched cohort*	PS-trimmed cohort	*Post hoc* PS stratification with extended PS
	Rivaroxaban (*n* = 3563) Apixaban (*n* = 3563)	Rivaroxaban (*n* = 3792) Apixaban (*n* = 4223)	Rivaroxaban (*n* = 3787) Apixaban (*n* = 4192)
Composite outcome	1.01 (0.82–1.25)	1.04 (0.86–1.26)	1.05 (0.87–1.28)
Stroke	0.78 (0.52–1.19)	0.83 (0.57–1.21)	0.84 (0.58–1.22)
Major bleeding	1.54 (1.14–2.08)	1.61 (1.22–2.12)	1.73 (1.30–2.28)
Myocardial infarction	0.96 (0.63–1.45)	0.97 (0.67–1.39)	0.98 (0.68–1.41)
All-cause death	0.97 (0.80–1.19)	1.06 (0.89–1.28)	1.07 (0.89–1.29)

*PS* propensity score; *CI* confidence interval

Variables that were selected for the primary analysis and the PS adjustment analysis can be found in the Supplemental material

^*^Unbalanced covariates: previous oral anticoagulant use within 3 months

#### PS-matched cohorts

The PS-matched set consisted of 3559 patients in each of the two treated groups, whose baseline characteristics are shown in Table 7B. Most were enrolled in Europe (51.9%). Of the rivaroxaban group, 76.2% of patients were prescribed 20 mg OD, and 20.7% received 15 mg OD; of the apixaban group, 81.8% of patients received 5 mg BID and 18.0% received 2.5 mg BID. The incidence rates for the key outcomes within the PS-matched cohort are shown in Table 8B.

Cox regression analysis of the PS-matched patient set, adjusted for unbalanced variables, revealed that treatment with rivaroxaban was associated with increased risk of major bleeding (HR: 1.54; 95% CI: 1.14–2.08; Table 9). Rates of stroke (HR: 0.78; 95% CI: 0.52–1.19), myocardial infarction (HR: 0.96; 95% CI: 0.63–1.45), all-cause death (HR 0.97; 95% CI: 0.80–1.19), and the composite outcome (HR: 1.01; 95% CI: 0.82–1.25) were similar for rivaroxaban and apixaban.* Post hoc* sensitivity analyses using an extended set of covariates in the propensity score confirmed the PS-matched analysis (Table 9).

## Discussion

In this large prospective study comparing various NOAC anticoagulants among patients with AF, the principal findings were that: (1) use of NOACs varied across world regions; (2) patients treated with dabigatran had a lower risk of major bleeding compared with rivaroxaban-treated patients, but risks of stroke, MI, mortality, and the composite outcome were similar with the two drugs; (3) there were similar risks of these events between patients treated with dabigatran or apixaban, and (4) patients treated with apixaban had a lower risk of major bleeding compared with rivaroxaban, but risks of stroke, MI, mortality, and the composite outcome were similar with the two drugs.

Geographic differences in prescribing patterns included less frequent use of apixaban in Asia and Latin America than in Europe and North America. Dabigatran was more often prescribed in Asian countries, while apixaban and rivaroxaban were more frequently employed in European patients. Some of these differences may reflect site selection, the timing of regulatory approvals in various markets, study enrollment timelines, economic/reimbursement conditions, or other variations in healthcare settings [21, 22].

While the study found broadly similar event rates with the individual NOACs, patients treated with rivaroxaban had a higher incidence rate of major bleeding compared with dabigatran. While in randomized trials all NOACs exhibited efficacy compared with warfarin, dosing was carried out differently. In the ARISTOTLE trial of apixaban [5] and the ROCKET-AF trial [4] of rivaroxaban, patients received standard doses unless reduced based on defined patient characteristics associated with increased drug exposure. In the RE-LY trial of dabigatran [6], two doses were administered in a randomized fashion without adjustment based on patient characteristics.

Observational studies have noted that NOAC dosing in clinical practice frequently does not align with labeled recommendations, with over 20% of patients prescribed the lower doses of rivaroxaban or apixaban in our analysis. We cannot ascertain whether dose selection for patients enrolled in GLORIA-AF was based on label-adherent criteria.

A meta-analysis of 12 observational case–control and cohort studies using multivariable or propensity score adjustment to estimate relative effects found comparable risks of stroke or systemic embolism with rivaroxaban vs apixaban and apixaban vs dabigatran in adults with AF [13]. In that analysis, apixaban had the most favorable safety profile, based on a lower rate of major bleeding than dabigatran and rivaroxaban [13].

A Danish nationwide study of 31,522 patients with AF comparing the effectiveness and safety of standard and reduced doses of NOACs found similar rates of stroke with standard and reduced NOAC doses. Rivaroxaban was associated with a higher risk of major bleeding than dabigatran or apixaban and dabigatran was associated with a lower risk of intracranial bleeding [14]. Among patients receiving reduced doses, apixaban was associated with a lower risk of gastrointestinal bleeding than the other NOACs [14]. In Norwegian and Korean cohorts, rivaroxaban was associated with a higher risk of major bleeding than dabigatran or apixaban [16, 17]. Whereas in the Global Anticoagulant Registry in the FIELD—Atrial Fibrillation (GARFIELD-AF) cohort of 25,551 anticoagulated patients with AF, the mortality rate was lower with NOACs than with VKA [23]. In that registry, therapy with dabigatran was associated with a lower risk of major bleeding than VKA (HR: 0.68; 95% CI: 0.47–0.98), while no substantial differences in the risk of bleeding were observed with FXa inhibitors compared with VKA (HR: 0.84; 95% CI: 0.63–1.12); but no separate analysis was performed for rivaroxaban and apixaban [23]. Data from these observational studies, suggesting an increased risk of major bleeding in rivaroxaban-treated patients compared with dabigatran and apixaban, and similar effectiveness with the various NOACs, are consistent with findings in GLORIA-AF.

### Limitations and strengths

This study is subject to potential confounding by factors not adjusted for in the analysis. The use of multiple imputation to address missing data for comparative analysis may result in bias if the missing-at-random assumption is violated. Despite the broad variety of sites and physician specialties in Asia, Europe, Latin America, and North America, over 50% of the study group was enrolled in Europe. Of note, dosing recommendations and criteria for dose reduction for NOACs differ across the world, and dabigatran 110 mg BID is not registered in the USA or Japan. Despite the large size of the GLORIA-AF cohort, the study size was not sufficient to permit comparison of NOACs based on dosage. Furthermore, the small number of patients prescribed edoxaban precluded its inclusion in comparative analyses.

Despite these limitations, GLORIA-AF is the first prospective global study of consecutive AF patients receiving anticoagulants in routine clinical practice for over 3 years. Regular follow-up visits with physicians, on-site monitoring, and data quality assurance standards, and the low proportion of patients for whom vital status was unavailable (4.7%) yielded high-quality, reliable data.

## Conclusions

In a routine clinical practice setting over 3 years, patients treated with dabigatran had a 41% lower risk of major bleeding compared with rivaroxaban and similar risks of stroke, MI and death. Relative to apixaban, dabigatran was associated with similar risks of stroke, major bleeding, MI, and all-cause death. Rivaroxaban relative to apixaban had an increased risk for major bleeding, however, similar risks for stroke, MI, and death.

## Supplementary Information

Below is the link to the electronic supplementary material.

Supplementary file1 (DOCX 75 KB)

Supplementary file2 (TIFF 167 KB)

Supplementary file3 (TIFF 168 KB)

Supplementary file3 (TIFF 161 KB)

## Data Availability

Clinical study documents (e.g., study report, study protocol, and statistical analysis plan) and participant clinical study data are available for sharing after publication of the primary manuscript in a peer-reviewed journal and if regulatory activities are complete and other criteria met, per the Boehringer Ingelheim Policy on Transparency and Publication of Clinical Study Data. Before providing access, documents will be examined, and, if necessary, redacted and the data will be de-identified, to protect the personal data of study participants and personnel, and to respect the boundaries of the informed consent of the study participants. Clinical study reports and related clinical documents can be requested. All requests will be governed by a document sharing agreement. Bona fide, qualified scientific and medical researchers might request access to de-identified, analysable participant clinical study data with corresponding documentation describing the structure and content of the datasets. On approval, and governed by a data-sharing agreement, data are shared in a secured data-access system for a period of 1 year, which might be extended on request. To request access to study data, see https://clinicalstudydatarequest.com
