# Correction to: Comparative effectiveness and safety of non-vitamin K antagonists for atrial fibrillation in clinical practice: GLORIA-AF Registry

**DOI:** 10.1007/s00392-022-02021-2

**Published:** 2022-04-06

**Authors:** Gregory Y. H. Lip, Agnieszka Kotalczyk, Christine Teutsch, Hans-Christoph Diener, Sergio J. Dubner, Jonathan L. Halperin, Chang-Sheng Ma, Kenneth J. Rothman, Sabrina Marler, Venkatesh Kumar Gurusamy, Menno V. Huisman, for the GLORIA-AF Investigators, Dzifa Wosornu Abban, Emad Aziz, Marica Bracic Kalan, Nasser Abdul, Luciano Marcelo Backes, Drew Bradman, Atilio Marcelo Abud, E. Badings, Donald Brautigam, Fran Adams, Ermentina Bagni, Nicolas Breton, Srinivas Addala, Seth H. Baker, P. J. A. M. Brouwers, Pedro Adragão, Richard Bala, Kevin Browne, Walter Ageno, Antonio Baldi, Jordi Bruguera Cortada, Rajesh Aggarwal, Shigenobu Bando, A. Bruni, Sergio Agosti, Subhash Banerjee, Claude Brunschwig, Piergiuseppe Agostoni, Alan Bank, Hervé Buathier, Francisco Aguilar, Gonzalo Barón Esquivias, Aurélie Buhl, Julio Aguilar Linares, Craig Barr, John Bullinga, Luis Aguinaga, Maria Bartlett, Jose Walter Cabrera, Jameel Ahmed, Vanja Basic Kes, Alberto Caccavo, Allessandro Aiello, Giovanni Baula, Shanglang Cai, Paul Ainsworth, Steffen Behrens, Sarah Caine, Jorge Roberto Aiub, Alan Bell, Leonardo Calò, Raed Al-Dallow, Raffaella Benedetti, Valeria Calvi, Lisa Alderson, Juan Benezet Mazuecos, Mauricio Camarillo Sánchez, Jorge Antonio Aldrete Velasco, Bouziane Benhalima, Rui Candeias, Dimitrios Alexopoulos, Jutta Bergler-Klein, Vincenzo Capuano, Fernando Alfonso Manterola, Jean-Baptiste Berneau, Alessandro Capucci, Pareed Aliyar, Richard A. Bernstein, Ronald Caputo, David Alonso, Percy Berrospi, Tatiana Cárdenas Rizo, Fernando Augusto Alves da Costa, Sergio Berti, Francisco Cardona, José Amado, Andrea Berz, Francisco Carlos da Costa Darrieux, Walid Amara, Elizabeth Best, Yan Carlos Duarte Vera, Mathieu Amelot, Paulo Bettencourt, Antonio Carolei, Nima Amjadi, Robert Betzu, Susana Carreño, Fabrizio Ammirati, Ravi Bhagwat, Paula Carvalho, Marianna Andrade, Luna Bhatta, Susanna Cary, Nabil Andrawis, Francesco Biscione, Gavino Casu, Giorgio Annoni, Giovanni Bisignani, Claudio Cavallini, Gerardo Ansalone, Toby Black, Guillaume Cayla, M. Kevin Ariani, Michael J. Bloch, Aldo Celentano, Juan Carlos Arias, Stephen Bloom, Tae-Joon Cha, Sébastien Armero, Edwin Blumberg, Kwang Soo Cha, Chander Arora, Mario Bo, Jei Keon Chae, Muhammad Shakil Aslam, Ellen Bøhmer, Kathrine Chalamidas, M. Asselman, Andreas Bollmann, Krishnan Challappa, Philippe Audouin, Maria Grazia Bongiorni, Sunil Prakash Chand, Charles Augenbraun, Giuseppe Boriani, Harinath Chandrashekar, S. Aydin, D. J. Boswijk, Ludovic Chartier, S. Aydin, Jochen Bott, Kausik Chatterjee, Ivaneta Ayryanova, Edo Bottacchi, Carlos Antero Chavez Ayala, Aamir Cheema, Gershan Davis, Rudolph Evonich, Amjad Cheema, Jean-Marc Davy, Oksana Evseeva, Lin Chen, Mark Dayer, Andrey Ezhov, Shih-Ann Chen, Marzia De Biasio, Raed Fahmy, Jyh Hong Chen, Silvana De Bonis, Quan Fang, Fu-Tien Chiang, Raffaele De Caterina, Ramin Farsad, Francesco Chiarella, Teresiano De Franceschi, Laurent Fauchier, Lin Chih-Chan, J. R. de Groot, Stefano Favale, Yong Keun Cho, José De Horta, Maxime Fayard, Jong-Il Choi, Axel De La Briolle, Jose Luis Fedele, Dong Ju Choi, Gilberto de la Pena Topete, Francesco Fedele, Guy Chouinard, Angelo Amato Vicenzo de Paola, Olga Fedorishina, Danny Hoi-Fan Chow, Weimar de Souza, Steven R. Fera, Dimitrios Chrysos, A. de Veer, Luis Gustavo Gomes Ferreira, Galina Chumakova, Luc De Wolf, Jorge Ferreira, Eduardo Julián José Roberto Chuquiure Valenzuela, Eric Decoulx, Claudio Ferri, Nicoleta Cindea Nica, Sasalu Deepak, Anna Ferrier, David J. Cislowski, Pascal Defaye, Hugo Ferro, Anthony Clay, Freddy Del-Carpio Munoz, Alexandra Finsen, Piers Clifford, Diana Delic Brkljacic, Brian First, Andrew Cohen, N. Joseph Deumite, Stuart Fischer, Michael Cohen, Silvia Di Legge, Catarina Fonseca, Serge Cohen, Igor Diemberger, Luísa Fonseca Almeida, Furio Colivicchi, Denise Dietz, Steven Forman, Ronan Collins, Pedro Dionísio, Brad Frandsen, Paolo Colonna, Qiang Dong, William French, Steve Compton, Fabio Rossi dos Santos, Keith Friedman, Derek Connolly, Elena Dotcheva, Athena Friese, Alberto Conti, Rami Doukky, Ana Gabriela Fruntelata, Gabriel Contreras Buenostro, Anthony D’Souza, Shigeru Fujii, Gregg Coodley, Simon Dubrey, Stefano Fumagalli, Martin Cooper, Xavier Ducrocq, Marta Fundamenski, Julian Coronel, Dmitry Dupljakov, Yutaka Furukawa, Giovanni Corso, Mauricio Duque, Matthias Gabelmann, Juan Cosín Sales, Dipankar Dutta, Nashwa Gabra, Yves Cottin, Nathalie Duvilla, Niels Gadsbøll, John Covalesky, A. Duygun, Michel Galinier, Aurel Cracan, Rainer Dziewas, Anders Gammelgaard, Filippo Crea, Charles B. Eaton, Priya Ganeshkumar, Peter Crean, William Eaves, Christopher Gans, James Crenshaw, L. A. Ebels-Tuinbeek, Antonio Garcia Quintana, Tina Cullen, Clifford Ehrlich, Olivier Gartenlaub, Harald Darius, Sabine Eichinger-Hasenauer, Achille Gaspardone, Patrick Dary, Steven J. Eisenberg, Conrad Genz, Olivier Dascotte, Adnan El Jabali, Frédéric Georger, Ira Dauber, Mahfouz El Shahawy, Jean-Louis Georges, Vicente Davalos, Mauro Esteves Hernandes, Steven Georgeson, Ruth Davies, Ana Etxeberria Izal, Evaldas Giedrimas, Mariusz Gierba, Tetsuya Haruna, Nabil Jarmukli, Ignacio Gil Ortega, Emil Hayek, Robert J. Jeanfreau, Eve Gillespie, Jeff Healey, Ronald D. Jenkins, Alberto Giniger, Steven Hearne, Carlos Jerjes Sánchez, Michael C. Giudici, Michael Heffernan, Javier Jimenez, Alexandros Gkotsis, Geir Heggelund, Robert Jobe, Taya V. Glotzer, J. A. Heijmeriks, Tomas Joen-Jakobsen, Joachim Gmehling, Maarten Hemels, Nicholas Jones, Jacek Gniot, I. Hendriks, Jose Carlos Moura Jorge, Peter Goethals, Sam Henein, Bernard Jouve, Seth Goldbarg, Sung-Ho Her, Byung Chun Jung, Ronald Goldberg, Paul Hermany, Kyung Tae Jung, Britta Goldmann, Jorge Eduardo Hernández Del Río, Werner Jung, Sergey Golitsyn, Yorihiko Higashino, Mikhail Kachkovskiy, Silvia Gómez, Michael Hill, Krystallenia Kafkala, Juan Gomez Mesa, Tetsuo Hisadome, Larisa Kalinina, Vicente Bertomeu Gonzalez, Eiji Hishida, Bernd Kallmünzer, Jesus Antonio Gonzalez Hermosillo, Etienne Hoffer, Farzan Kamali, Víctor Manuel González López, Matthew Hoghton, Takehiro Kamo, Hervé Gorka, Kui Hong, Priit Kampus, Charles Gornick, Suk keun Hong, Hisham Kashou, Diana Gorog, Stevie Horbach, Andreas Kastrup, Venkat Gottipaty, Masataka Horiuchi, Apostolos Katsivas, Pascal Goube, Yinglong Hou, Elizabeth Kaufman, Ioannis Goudevenos, Jeff Hsing, Kazuya Kawai, Brett Graham, Chi-Hung Huang, Kenji Kawajiri, G. Stephen Greer, David Huckins, John F. Kazmierski, Uwe Gremmler, Kathy Hughes, P. Keeling, Paul G. Grena, A. Huizinga, José Francisco Kerr Saraiva, Martin Grond, E. L. Hulsman, Galina Ketova, Edoardo Gronda, Kuo-Chun Hung, AJIT Singh Khaira, Gerian Grönefeld, Gyo-Seung Hwang, Aleksey Khripun, Xiang Gu, Margaret Ikpoh, Doo-Il Kim, Ivett Guadalupe Torres Torres, Davide Imberti, Young Hoon Kim, Gabriele Guardigli, Hüseyin Ince, Nam Ho Kim, Carolina Guevara, Ciro Indolfi, Dae Kyeong Kim, Alexandre Guignier, Shujiro Inoue, Jeong Su Kim, Michele Gulizia, Didier Irles, June Soo Kim, Michael Gumbley, Harukazu Iseki, Ki Seok Kim, Albrecht Günther, C. Noah Israel, Jin bae Kim, Andrew Ha, Bruce Iteld, Elena Kinova, Georgios Hahalis, Venkat Iyer, Alexander Klein, Joseph Hakas, Ewart Jackson-Voyzey, James J. Kmetzo, Christian Hall, Naseem Jaffrani, G. Larsen Kneller, Bing Han, Frank Jäger, Aleksandar Knezevic, Seongwook Han, Martin James, Su Mei Angela Koh, Joe Hargrove, Sung-Won Jang, Shunichi Koide, David Hargroves, Nicolas Jaramillo, Anastasios Kollias, J. A. Kooistra, Weihua Li, John McClure, Jay Koons, Xiaoming Li, Terry McCormack, Martin Koschutnik, Christhoh Lichy, William McGarity, William J. Kostis, Ira Lieber, Hugh McIntyre, Dragan Kovacic, Ramon Horacio Limon Rodriguez, Brent McLaurin, Jacek Kowalczyk, Hailong Lin, Feliz Alvaro, Medina Palomino, Natalya Koziolova, Gregory Y. H. Lip, Francesco Melandri, Peter Kraft, Feng Liu, Hiroshi Meno, Johannes A. Kragten, Hengliang Liu, Dhananjai Menzies, Mori Krantz, Guillermo Llamas Esperon, Marco Mercader, Lars Krause, Nassip Llerena Navarro, Christian Meyer, B. J. Krenning, Eric Lo, Beat J. Meyer, F. Krikke, Sergiy Lokshyn, Jacek Miarka, Z. Kromhout, Amador López, Frank Mibach, Waldemar Krysiak, José Luís López-Sendón, Dominik Michalski, Priya Kumar, Adalberto Menezes Lorga Filho, Patrik Michel, Thomas Kümler, Richard S. Lorraine, Rami Mihail Chreih, Malte Kuniss, Carlos Alberto Luengas, Alberto Luengas, Ghiath Mikdadi, Jen-Yuan Kuo, Robert Luke, Milan Mikus, Achim Küppers, Ming Luo, Davor Milicic, Karla Kurrelmeyer, Steven Lupovitch, Constantin Militaru, Choong Hwan Kwak, Philippe Lyrer, Sedi Minaie, Bénédicte Laboulle, Changsheng Ma, Bogdan Minescu, Arthur Labovitz, Genshan Ma, Iveta Mintale, Wen Ter Lai, Irene Madariaga, Tristan Mirault, Andy Lam, Koji Maeno, Michael J. Mirro, Yat Yin Lam, Dominique Magnin, Dinesh Mistry, Fernando Lanas Zanetti, Gustavo Maid, Nicoleta Violeta Miu, Charles Landau, Sumeet K. Mainigi, Naomasa Miyamoto, Giancarlo Landini, Konstantinos Makaritsis, Tiziano Moccetti, Estêvão Lanna Figueiredo, Rohit Malhotra, Akber Mohammed, Torben Larsen, Rickey Manning, Azlisham Mohd Nor, Karine Lavandier, Athanasios Manolis, Michael Mollerus, Jessica LeBlanc, Helard Andres Manrique Hurtado, Giulio Molon, Moon Hyoung Lee, Ioannis Mantas, Sergio Mondillo, Chang-Hoon Lee, Fernando Manzur Jattin, Patrícia Moniz, John Lehman, Vicky Maqueda, Lluis Mont, Ana Leitão, Niccolo Marchionni, Vicente Montagud, Nicolas Lellouche, Francisco Marin Ortuno, Oscar Montaña, Malgorzata Lelonek, Antonio Martín Santana, Cristina Monti, Radoslaw Lenarczyk, Jorge Martinez, Luciano Moretti, T. Lenderink, Petra Maskova, Kiyoo Mori, Salvador León González, Norberto Matadamas Hernandez, Andrew Moriarty, Peter Leong-Sit, Katsuhiro Matsuda, Jacek Morka, Matthias Leschke, Tillmann Maurer, Luigi Moschini, Nicolas Ley, Ciro Mauro, Nikitas Moschos, Zhanquan Li, Erik May, Andreas Mügge, Xiaodong Li, Nolan Mayer, Thomas J. Mulhearn, Carmen Muresan, Eena Padayattil Jose, Dalton Bertolim Précoma, Michela Muriago, Francisco Gerardo Padilla Padilla, Alessandro Prelle, Wlodzimierz Musial, Victoria Padilla Rios, John Prodafikas, Carl W. Musser, Giuseppe Pajes, Konstantin Protasov, Francesco Musumeci, A. Shekhar Pandey, Maurice Pye, Thuraia Nageh, Gaetano Paparella, Zhaohui Qiu, Hidemitsu Nakagawa, F. Paris, Jean-Michel Quedillac, Yuichiro Nakamura, Hyung Wook Park, Dimitar Raev, Toru Nakayama, Jong Sung Park, Carlos Antonio Raffo Grado, Gi-Byoung Nam, Fragkiskos Parthenakis, Sidiqullah Rahimi, Michele Nanna, Enrico Passamonti, Arturo Raisaro, Indira Natarajan, Rajesh J. Patel, Bhola Rama, Hemal M. Nayak, Jaydutt Patel, Ricardo Ramos, Stefan Naydenov, Mehool Patel, Maria Ranieri, Jurica Nazlić, Janice Patrick, Nuno Raposo, Alexandru Cristian Nechita, Ricardo Pavón Jimenez, Eric Rashba, Libor Nechvatal, Analía Paz, Ursula Rauch-Kroehnert, Sandra Adela Negron, Vittorio Pengo, Ramakota Reddy, James Neiman, William Pentz, Giulia Renda, Fernando Carvalho Neuenschwander, Beatriz Pérez, Shabbir Reza, David Neves, Alma Minerva Pérez Ríos, Luigi Ria, Anna Neykova, Alejandro Pérez-Cabezas, Dimitrios Richter, Ricardo Nicolás Miguel, Richard Perlman, Hans Rickli, George Nijmeh, Viktor Persic, Werner Rieker, Alexey Nizov, Francesco Perticone, Tomas Ripolil Vera, Rodrigo Noronha Campos, Terri K. Peters, Luiz Eduardo Ritt, Janko Nossan, Sanjiv Petkar, Douglas Roberts, Tatiana Novikova, Luis Felipe Pezo, Ignacio Rodriguez Briones, Ewa Nowalany-Kozielska, Christian Pflücke, Aldo Edwin Rodriguez Escudero, Emmanuel Nsah, David N. Pham, Carlos Rodríguez Pascual, Juan Carlos Nunez Fragoso, Roland T. Phillips, Mark Roman, Svetlana Nurgalieva, Stephen Phlaum, Francesco Romeo, Dieter Nuyens, Denis Pieters, E. Ronner, Ole Nyvad, Julien Pineau, Jean-Francois Roux, Manuel Odin de Los Rios Ibarra, Arnold Pinter, Nadezda Rozkova, Philip O’Donnell, Fausto Pinto, Miroslav Rubacek, Martin O’Donnell, R. Pisters, Frank Rubalcava, Seil Oh, Nediljko Pivac, Andrea M. Russo, Yong Seog Oh, Darko Pocanic, Matthieu Pierre Rutgers, Dongjin Oh, Cristian Podoleanu, Karin Rybak, Gilles O‘Hara, Alessandro Politano, Samir Said, Kostas Oikonomou, Zdravka Poljakovic, Tamotsu Sakamoto, Claudia Olivares, Stewart Pollock, Abraham Salacata, Richard Oliver, Jose Polo Garcéa, Adrien Salem, Rafael Olvera Ruiz, Holger Poppert, Rafael Salguero Bodes, Christoforos Olympios, Maurizio Porcu, Marco A. Saltzman, Anna Omaszuk-Kazberuk, Antonio Pose Reino, Alessandro Salvioni, Joaquín Osca Asensi, Neeraj Prasad, Gregorio Sanchez Vallejo, Marcelo Sanmartín Fernández, Adam Sokal, Tian Ming Tu, Wladmir Faustino Saporito, Yannie Soo Oi Yan, Ype Tuininga, Kesari Sarikonda, Rodolfo Sotolongo, Minang Turakhia, Taishi Sasaoka, Olga Ferreira de Souza, Samir Turk, Hamdi Sati, Jon Arne Sparby, Wayne Turner, Irina Savelieva, Jindrich Spinar, Arnljot Tveit, Pierre-Jean Scala, David Sprigings, Richard Tytus, Peter Schellinger, Alex C. Spyropoulos, C. Valadão, Carlos Scherr, Dimitrios Stakos, P. F. M. M. van Bergen, Lisa Schmitz, Clemens Steinwender, Philippe van de Borne, Karl-Heinz Schmitz, Georgios Stergiou, B. J. van den Berg, Bettina Schmitz, Ian Stiell, C. van der Zwaan, Teresa Schnabel, Marcus Stoddard, M. Van Eck, Steffen Schnupp, Anastas Stoikov, Peter Vanacker, Peter Schoeniger, Witold Streb, Dimo Vasilev, Norbert Schön, Ioannis Styliadis, Vasileios Vasilikos, Peter Schwimmbeck, Guohai Su, Maxim Vasilyev, Clare Seamark, Xi Su, Srikar Veerareddy, Greg Searles, Wanda Sudnik, Mario Vega Miño, Karl-Heinz Seidl, Kai Sukles, Asok Venkataraman, Barry Seidman, Xiaofei Sun, Paolo Verdecchia, Jaroslaw Sek, H. Swart, Francesco Versaci, Lakshmanan Sekaran, Janko Szavits-Nossan, Ernst Günter Vester, Carlo Serrati, Jens Taggeselle, Hubert Vial, Neerav Shah, Yuichiro Takagi, Jason Victory, Vinay Shah, Amrit Pal Singh Takhar, Alejandro Villamil, Anil Shah, Angelika Tamm, Marc Vincent, Shujahat Shah, Katsumi Tanaka, Anthony Vlastaris, Vijay Kumar Sharma, Tanyanan Tanawuttiwat, Jürgen vom Dahl, Louise Shaw, Sherman Tang, Kishor Vora, Khalid H. Sheikh, Aylmer Tang, Robert B. Vranian, Naruhito Shimizu, Giovanni Tarsi, Paul Wakefield, Hideki Shimomura, Tiziana Tassinari, Ningfu Wang, Dong-Gu Shin, Ashis Tayal, Mingsheng Wang, Eun-Seok Shin, Muzahir Tayebjee, Xinhua Wang, Junya Shite, J. M. ten Berg, Feng Wang, Gerolamo Sibilio, Dan Tesloianu, Tian Wang, Frank Silver, Salem H. K. The, Alberta L. Warner, Iveta Sime, Dierk Thomas, Kouki Watanabe, Tim A. Simmers, Serge Timsit, Jeanne Wei, Narendra Singh, Tetsuya Tobaru, Christian Weimar, Peter Siostrzonek, Andrzej R. Tomasik., Stanislav Weiner, Didier Smadja, Mikhail Torosoff, Renate Weinrich, David W. Smith, Emmanuel Touze, Ming-Shien Wen, Marcelo Snitman, Elina Trendafilova, Marcus Wiemer, Dario Sobral Filho, W. Kevin Tsai, Preben Wiggers, Hassan Soda, Hung Fat Tse, Andreas Wilke, Carl Sofley, Hiroshi Tsutsui, David Williams, Marcus L. Williams, Ping Yen Bryan Yan, Ping Zhang, Bernhard Witzenbichler, Tianlun Yang, Jun Zhang, Brian Wong, Jing Yao, Shui Ping Zhao, Ka Sing Lawrence Wong, Kuo-Ho Yeh, Yujie Zhao, Beata Wozakowska-Kaplon, Wei Hsian Yin, Zhichen Zhao, Shulin Wu, Yoto Yotov, Yang Zheng, Richard C. Wu, Ralf Zahn, Jing Zhou, Silke Wunderlich, Stuart Zarich, Sergio Zimmermann, Nell Wyatt, Sergei Zenin, Andrea Zini, John Wylie, Elisabeth Louise Zeuthen, Steven Zizzo, Yong Xu, Huanyi Zhang, Wenxia Zong, Xiangdong Xu, Donghui Zhang, LSteven Zukerman, Hiroki Yamanoue, Xingwei Zhang, Takeshi Yamashita

**Affiliations:** 1 Liverpool Centre for Cardiovascular Science, University of Liverpool and Liverpool Heart and Chest Hospital, Liverpool, UK; 2 Department of Cardiology, Congenital Heart Diseases and Electrotherapy, Medical University of Silesia, Silesian Centre for Heart Diseases, Zabrze, Poland; 3 Aalborg Thrombosis Research Unit, Department of Clinical Medicine, Aalborg University, Aalborg, Denmark; 4 Department of Cardiometabolism and Respiratory Medicine, Boehringer Ingelheim International GmbH, Ingelheim, Germany; 5 Department of Neuroepidemiology, Institute for Medical Informatics, Biometry and Epidemiology (IMIBE), University Duisburg-Essen, Essen, Germany; 6 Clínica y Maternidad Suizo Argentina, Buenos Aires, Argentina; 7 The Cardiovascular Institute, Mount Sinai Medical Center, New York, NY USA; 8 Cardiology Department, Atrial Fibrillation Center, Beijing Anzhen Hospital, Capital Medical University, Beijing, China; 9 RTI Health Solutions, Research Triangle Park, NC USA; 10 Biostatistics and Data Sciences, Boehringer Ingelheim Pharmaceuticals, Inc, Ridgefield, CT USA; 11 Global Epidemiology, Boehringer Ingelheim International GmbH, Ingelheim, Germany; 12 Department of Thrombosis and Hemostasis, Leiden University Medical Center, Leiden, The Netherlands

## Correction to: Clinical Research in Cardiology 10.1007/s00392-022-01996-2

In this article, the name of the GLORIA-AF investigator Anastasios Kollias was given incorrectly as Athanasios Kollias in the Acknowledgements. The original article has been corrected.

