# Supplementary material for: Comparative effectiveness and safety of non-vitamin K antagonists for atrial fibrillation in clinical practice: GLORIA-AF Registry
Source: Clin Res Cardiol. 2022 Mar 16;111(5):560–73. doi: 10.1007/s00392-022-01996-2 (PMC9054878; doi:10.1007/s00392-022-01996-2)
Supplement: Supplementary file 1 — Supplementary file1 (DOCX 75 KB) [file 392_2022_1996_MOESM1_ESM.docx]

**Online Resource**

**Comparative effectiveness and safety of non-vitamin K antagonists for atrial fibrillation in clinical practice: GLORIA-AF Registry**

Gregory Y. H. Lip (ORCID: 0000-0002-7566-1626); Agnieszka Kotalczyk (ORCID: 0000-0002-6762-3187), Christine Teutsch, Hans-Christoph Diener, Sergio J. Dubner, Jonathan L. Halperin, Chang-Sheng Ma, Kenneth J. Rothman, Sabrina Marler, Venkatesh Kumar Gurusamy and Menno V. Huisman, for the GLORIA-AF Investigators

**CONTENTS**

**INVESTIGATOR LIST**

**DATA SHARING STATEMENT**

**METHODS**

**1. Complete Inclusion/Exclusion Criteria**

**2. Definition of Life-Threatening Bleeds**

**3. Further Details of the Imputation Method for Missing Data**

**4. Further Details of the Propensity Score Trimmed and Matched Patient Sets**

**5. Further Details of the Additional Covariate Selection Procedure for the Multivariable Cox Regression Model**

**6. Further Details of Sensitivity Analyses**

# FIGURES

**Figure 1. Propensity score overlap for dabigatran vs rivaroxaban within the regions**.

# Figure 2. Propensity score overlap for dabigatran vs apixaban within the regions.

# Figure 3. Propensity score overlap for rivaroxaban vs apixaban within the regions.

# TABLES

**Table 1. Variables used in Cox and PS models**

# Table 2. The baseline characteristics of the eligible patients treated with dabigatran, rivaroxaban and apixaban.

**INVESTIGATOR LIST**

**Investigators participating in GLORIA-AF Phase III**

| Dzifa Wosornu Abban | Emad Aziz | Marica Bracic Kalan |
| --- | --- | --- |
| Nasser Abdul | Luciano Marcelo Backes | Drew Bradman |
| Atilio Marcelo Abud | E. Badings | Donald Brautigam |
| Fran Adams | Ermentina Bagni | Nicolas Breton |
| Srinivas Addala | Seth H. Baker | P.J.A.M. Brouwers |
| Pedro Adragão | Richard Bala | Kevin Browne |
| Walter Ageno | Antonio Baldi | Jordi Bruguera Cortada |
| Rajesh Aggarwal | Shigenobu Bando | A. Bruni |
| Sergio Agosti | Subhash Banerjee | Claude Brunschwig |
| Piergiuseppe Agostoni | Alan Bank | Hervé Buathier |
| Francisco Aguilar | Gonzalo Barón Esquivias | Aurélie Buhl |
| Julio Aguilar Linares | Craig Barr | John Bullinga |
| Luis Aguinaga | Maria Bartlett | Jose Walter Cabrera |
| Jameel Ahmed | Vanja Basic Kes | Alberto Caccavo |
| Allessandro Aiello | Giovanni Baula | Shanglang Cai |
| Paul Ainsworth | Steffen Behrens | Sarah Caine |
| Jorge Roberto Aiub | Alan Bell | Leonardo Calò |
| Raed Al-Dallow | Raffaella Benedetti | Valeria Calvi |
| Lisa Alderson | Juan Benezet Mazuecos | Mauricio Camarillo Sánchez |
| Jorge Antonio Aldrete Velasco | Bouziane Benhalima | Rui Candeias |
| Dimitrios Alexopoulos | Jutta Bergler-Klein | Vincenzo Capuano |
| Fernando Alfonso Manterola | Jean-Baptiste Berneau | Alessandro Capucci |
| Pareed Aliyar | Richard A. Bernstein | Ronald Caputo |
| David Alonso | Percy Berrospi | Tatiana Cárdenas Rizo |
| Fernando Augusto Alves da Costa | Sergio Berti | Francisco Cardona |
| José Amado | Andrea Berz | Francisco Carlos da Costa Darrieux |
| Walid Amara | Elizabeth Best | Yan Carlos Duarte Vera |
| Mathieu Amelot | Paulo Bettencourt | Antonio Carolei |
| Nima Amjadi | Robert Betzu | Susana Carreño |
| Fabrizio Ammirati | Ravi Bhagwat | Paula Carvalho |
| Marianna Andrade | Luna Bhatta | Susanna Cary |
| Nabil Andrawis | Francesco Biscione | Gavino Casu |
| Giorgio Annoni | Giovanni Bisignani | Claudio Cavallini |
| Gerardo Ansalone | Toby Black | Guillaume Cayla |
| M.Kevin Ariani | Michael J. Bloch | Aldo Celentano |
| Juan Carlos Arias | Stephen Bloom | Tae-Joon Cha |
| Sébastien Armero | Edwin Blumberg | Kwang Soo Cha |
| Chander Arora | Mario Bo | Jei Keon Chae |
| Muhammad Shakil Aslam | Ellen Bøhmer | Kathrine Chalamidas |
| M. Asselman | Andreas Bollmann | Krishnan Challappa |
| Philippe Audouin | Maria Grazia Bongiorni | Sunil Prakash Chand |
| Charles Augenbraun | Giuseppe Boriani | Harinath Chandrashekar |
| S. Aydin | D.J. Boswijk | Ludovic Chartier |
| S. Aydin | Jochen Bott | Kausik Chatterjee |
| Ivaneta Ayryanova | Edo Bottacchi | Carlos Antero Chavez Ayala |

| Aamir Cheema | Gershan Davis | Rudolph Evonich III |
| --- | --- | --- |
| Amjad Cheema | Jean-Marc Davy | Oksana Evseeva |
| Lin Chen | Mark Dayer | Andrey Ezhov |
| Shih-Ann Chen | Marzia De Biasio | Raed Fahmy |
| Jyh Hong Chen | Silvana De Bonis | Quan Fang |
| Fu-Tien Chiang | Raffaele De Caterina | Ramin Farsad |
| Francesco Chiarella | Teresiano De Franceschi | Laurent Fauchier |
| Lin Chih-Chan | J.R. de Groot | Stefano Favale |
| Yong Keun Cho | José De Horta | Maxime Fayard |
| Jong-Il Choi | Axel De La Briolle | Jose Luis Fedele |
| Dong Ju Choi | Gilberto de la Pena Topete | Francesco Fedele |
| Guy Chouinard | Angelo Amato Vicenzo de Paola | Olga Fedorishina |
| Danny Hoi-Fan Chow | Weimar de Souza | Steven R. Fera |
| Dimitrios Chrysos | A. de Veer | Luis Gustavo Gomes Ferreira |
| Galina Chumakova | Luc De Wolf | Jorge Ferreira |
| Eduardo Julián José Roberto Chuquiure Valenzuela | Eric Decoulx | Claudio Ferri |
| Nicoleta Cindea Nica | Sasalu Deepak | Anna Ferrier |
| David J. Cislowski | Pascal Defaye | Hugo Ferro |
| Anthony Clay | Freddy Del-Carpio Munoz | Alexandra Finsen |
| Piers Clifford | Diana Delic Brkljacic | Brian First |
| Andrew Cohen | N. Joseph Deumite | Stuart Fischer |
| Michael Cohen | Silvia Di Legge | Catarina Fonseca |
| Serge Cohen | Igor Diemberger | Luísa Fonseca Almeida |
| Furio Colivicchi | Denise Dietz | Steven Forman |
| Ronan Collins | Pedro Dionísio | Brad Frandsen |
| Paolo Colonna | Qiang Dong | William French |
| Steve Compton | Fabio Rossi dos Santos | Keith Friedman |
| Derek Connolly | Elena Dotcheva | Athena Friese |
| Alberto Conti | Rami Doukky | Ana Gabriela Fruntelata |
| Gabriel Contreras Buenostro | Anthony D'Souza | Shigeru Fujii |
| Gregg Coodley | Simon Dubrey | Stefano Fumagalli |
| Martin Cooper | Xavier Ducrocq | Marta Fundamenski |
| Julian Coronel | Dmitry Dupljakov | Yutaka Furukawa |
| Giovanni Corso | Mauricio Duque | Matthias Gabelmann |
| Juan Cosín Sales | Dipankar Dutta | Nashwa Gabra |
| Yves Cottin | Nathalie Duvilla | Niels Gadsbøll |
| John Covalesky | A. Duygun | Michel Galinier |
| Aurel Cracan | Rainer Dziewas | Anders Gammelgaard |
| Filippo Crea | Charles B. Eaton | Priya Ganeshkumar |
| Peter Crean | William Eaves | Christopher Gans |
| James Crenshaw | L.A Ebels-Tuinbeek | Antonio Garcia Quintana |
| Tina Cullen | Clifford Ehrlich | Olivier Gartenlaub |
| Harald Darius | Sabine Eichinger-Hasenauer | Achille Gaspardone |
| Patrick Dary | Steven J. Eisenberg | Conrad Genz |
| Olivier Dascotte | Adnan El Jabali | Frédéric Georger |
| Ira Dauber | Mahfouz El Shahawy | Jean-Louis Georges |
| Vicente Davalos | Mauro Esteves Hernandes | Steven Georgeson |
| Ruth Davies | Ana Etxeberria Izal | Evaldas Giedrimas |

| Mariusz Gierba | Tetsuya Haruna | Nabil Jarmukli |
| --- | --- | --- |
| Ignacio Gil Ortega | Emil Hayek | Robert J. Jeanfreau |
| Eve Gillespie | Jeff Healey | Ronald D. Jenkins |
| Alberto Giniger | Steven Hearne | Carlos Jerjes Sánchez |
| Michael C. Giudici | Michael Heffernan | Javier Jimenez |
| Alexandros Gkotsis | Geir Heggelund | Robert Jobe |
| Taya V. Glotzer | J.A. Heijmeriks | Tomas Joen-Jakobsen |
| Joachim Gmehling | Maarten Hemels | Nicholas Jones |
| Jacek Gniot | I. Hendriks | Jose Carlos Moura Jorge |
| Peter Goethals | Sam Henein | Bernard Jouve |
| Seth Goldbarg | Sung-Ho Her | Byung Chun Jung |
| Ronald Goldberg | Paul Hermany | Kyung Tae Jung |
| Britta Goldmann | Jorge Eduardo Hernández Del Río | Werner Jung |
| Sergey Golitsyn | Yorihiko Higashino | Mikhail Kachkovskiy |
| Silvia Gómez | Michael Hill | Krystallenia Kafkala |
| Juan Gomez Mesa | Tetsuo Hisadome | Larisa Kalinina |
| Vicente Bertomeu Gonzalez | Eiji Hishida | Bernd Kallmünzer |
| Jesus Antonio Gonzalez Hermosillo | Etienne Hoffer | Farzan Kamali |
| Víctor Manuel González López | Matthew Hoghton | Takehiro Kamo |
| Hervé Gorka | Kui Hong | Priit Kampus |
| Charles Gornick | Suk keun Hong | Hisham Kashou |
| Diana Gorog | Stevie Horbach | Andreas Kastrup |
| Venkat Gottipaty | Masataka Horiuchi | Apostolos Katsivas |
| Pascal Goube | Yinglong Hou | Elizabeth Kaufman |
| Ioannis Goudevenos | Jeff Hsing | Kazuya Kawai |
| Brett Graham | Chi-Hung Huang | Kenji Kawajiri |
| G. Stephen Greer | David Huckins | John F. Kazmierski |
| Uwe Gremmler | Kathy Hughes | P Keeling |
| Paul G. Grena | A. Huizinga | José Francisco Kerr Saraiva |
| Martin Grond | E.L. Hulsman | Galina Ketova |
| Edoardo Gronda | Kuo-Chun Hung | AJIT Singh Khaira |
| Gerian Grönefeld | Gyo-Seung Hwang | Aleksey Khripun |
| Xiang Gu | Margaret Ikpoh | Doo-Il Kim |
| Ivett Guadalupe Torres Torres | Davide Imberti | Young Hoon Kim |
| Gabriele Guardigli | Hüseyin Ince | Nam Ho Kim |
| Carolina Guevara | Ciro Indolfi | Dae Kyeong Kim |
| Alexandre Guignier | Shujiro Inoue | Jeong Su Kim |
| Michele Gulizia | Didier Irles | June Soo Kim |
| Michael Gumbley | Harukazu Iseki | Ki Seok Kim |
| Albrecht Günther | C. Noah Israel | Jin bae Kim |
| Andrew Ha | Bruce Iteld | Elena Kinova |
| Georgios Hahalis | Venkat Iyer | Alexander Klein |
| Joseph Hakas | Ewart Jackson-Voyzey | James J. Kmetzo |
| Christian Hall | Naseem Jaffrani | G. Larsen Kneller |
| Bing Han | Frank Jäger | Aleksandar Knezevic |
| Seongwook Han | Martin James | Su Mei Angela Koh |
| Joe Hargrove | Sung-Won Jang | Shunichi Koide |
| David Hargroves | Nicolas Jaramillo | Athanasios Kollias |

| J.A. Kooistra | Weihua Li | John McClure |
| --- | --- | --- |
| Jay Koons | Xiaoming Li | Terry McCormack |
| Martin Koschutnik | Christhoh Lichy | William McGarity |
| William J. Kostis | Ira Lieber | Hugh McIntyre |
| Dragan Kovacic | Ramon Horacio Limon Rodriguez | Brent McLaurin |
| Jacek Kowalczyk | Hailong Lin | Feliz Alvaro Medina Palomino |
| Natalya Koziolova | Gregory Y. H. Lip | Francesco Melandri |
| Peter Kraft | Feng Liu | Hiroshi Meno |
| Johannes A. Kragten | Hengliang Liu | Dhananjai Menzies |
| Mori Krantz | Guillermo Llamas Esperon | Marco Mercader |
| Lars Krause | Nassip Llerena Navarro | Christian Meyer |
| B.J. Krenning | Eric Lo | Beat j. Meyer |
| F. Krikke | Sergiy Lokshyn | Jacek Miarka |
| Z. Kromhout | Amador López | Frank Mibach |
| Waldemar Krysiak | José Luís López-Sendón | Dominik Michalski |
| Priya Kumar | Adalberto Menezes Lorga Filho | Patrik Michel |
| Thomas Kümler | Richard S. Lorraine | Rami Mihail Chreih |
| Malte Kuniss | Carlos Alberto Luengas | Ghiath Mikdadi |
| Jen-Yuan Kuo | Robert Luke | Milan Mikus |
| Achim Küppers | Ming Luo | Davor Milicic |
| Karla Kurrelmeyer | Steven Lupovitch | Constantin Militaru |
| Choong Hwan Kwak | Philippe Lyrer | Sedi Minaie |
| Bénédicte Laboulle | Changsheng Ma | Bogdan Minescu |
| Arthur Labovitz | Genshan Ma | Iveta Mintale |
| Wen Ter Lai | Irene Madariaga | Tristan Mirault |
| Andy Lam | Koji Maeno | Michael J. Mirro |
| Yat Yin Lam | Dominique Magnin | Dinesh Mistry |
| Fernando Lanas Zanetti | Gustavo Maid | Nicoleta Violeta Miu |
| Charles Landau | Sumeet K. Mainigi | Naomasa Miyamoto |
| Giancarlo Landini | Konstantinos Makaritsis | Tiziano Moccetti |
| Estêvão Lanna Figueiredo | Rohit Malhotra | Akber Mohammed |
| Torben Larsen | Rickey Manning | Azlisham Mohd Nor |
| Karine Lavandier | Athanasios Manolis | Michael Mollerus |
| Jessica LeBlanc | Helard Andres Manrique Hurtado | Giulio Molon |
| Moon Hyoung Lee | Ioannis Mantas | Sergio Mondillo |
| Chang-Hoon Lee | Fernando Manzur Jattin | Patrícia Moniz |
| John Lehman | Vicky Maqueda | Lluis Mont |
| Ana Leitão | Niccolo Marchionni | Vicente Montagud |
| Nicolas Lellouche | Francisco Marin Ortuno | Oscar Montaña |
| Malgorzata Lelonek | Antonio Martín Santana | Cristina Monti |
| Radoslaw Lenarczyk | Jorge Martinez | Luciano Moretti |
| T. Lenderink | Petra Maskova | Kiyoo Mori |
| Salvador León González | Norberto Matadamas Hernandez | Andrew Moriarty |
| Peter Leong-Sit | Katsuhiro Matsuda | Jacek Morka |
| Matthias Leschke | Tillmann Maurer | Luigi Moschini |
| Nicolas Ley | Ciro Mauro | Nikitas Moschos |
| Zhanquan Li | Erik May | Andreas Mügge |
| Xiaodong Li | Nolan Mayer | Thomas J. Mulhearn |

| Carmen Muresan | eena Padayattil jose | Dalton Bertolim Précoma |
| --- | --- | --- |
| Michela Muriago | Francisco Gerardo Padilla Padilla | Alessandro Prelle |
| Wlodzimierz Musial | Victoria Padilla Rios | John Prodafikas |
| Carl W. Musser | Giuseppe Pajes | Konstantin Protasov |
| Francesco Musumeci | A. Shekhar Pandey | Maurice Pye |
| Thuraia Nageh | Gaetano Paparella | Zhaohui Qiu |
| Hidemitsu Nakagawa | F Paris | Jean-Michel Quedillac |
| Yuichiro Nakamura | Hyung Wook Park | Dimitar Raev |
| Toru Nakayama | Jong Sung Park | Carlos Antonio Raffo Grado |
| Gi-Byoung Nam | Fragkiskos Parthenakis | Sidiqullah Rahimi |
| Michele Nanna | Enrico Passamonti | Arturo Raisaro |
| Indira Natarajan | Rajesh J. Patel | Bhola Rama |
| Hemal M. Nayak | Jaydutt Patel | Ricardo Ramos |
| Stefan Naydenov | Mehool Patel | Maria Ranieri |
| Jurica Nazlić | Janice Patrick | Nuno Raposo |
| Alexandru Cristian Nechita | Ricardo Pavón Jimenez | Eric Rashba |
| Libor Nechvatal | Analía Paz | Ursula Rauch-Kroehnert |
| Sandra Adela Negron | Vittorio Pengo | Ramakota Reddy |
| James Neiman | William Pentz | Giulia Renda |
| Fernando Carvalho Neuenschwander | Beatriz Pérez | Shabbir Reza |
| David Neves | Alma Minerva Pérez Ríos | Luigi Ria |
| Anna Neykova | Alejandro Pérez-Cabezas | Dimitrios Richter |
| Ricardo Nicolás Miguel | Richard Perlman | Hans Rickli |
| George Nijmeh | Viktor Persic | Werner Rieker |
| Alexey Nizov | Francesco Perticone | Tomas Ripolil Vera |
| Rodrigo Noronha Campos | Terri K. Peters | Luiz Eduardo Ritt |
| Janko Nossan | Sanjiv Petkar | Douglas Roberts |
| Tatiana Novikova | Luis Felipe Pezo | Ignacio Rodriguez Briones |
| Ewa Nowalany-Kozielska | Christian Pflücke | Aldo Edwin Rodriguez Escudero |
| Emmanuel Nsah | David N. Pham | Carlos Rodríguez Pascual |
| Juan Carlos Nunez Fragoso | Roland T. Phillips | Mark Roman |
| Svetlana Nurgalieva | Stephen Phlaum | Francesco Romeo |
| Dieter Nuyens | Denis Pieters | E. Ronner |
| Ole Nyvad | Julien Pineau | Jean-Francois Roux |
| Manuel Odin de Los Rios Ibarra | Arnold Pinter | Nadezda Rozkova |
| Philip O'Donnell | Fausto Pinto | Miroslav Rubacek |
| Martin O'Donnell | R. Pisters | Frank Rubalcava |
| Seil Oh | Nediljko Pivac | Andrea M. Russo |
| Yong Seog Oh | Darko Pocanic | Matthieu Pierre Rutgers |
| Dongjin Oh | Cristian Podoleanu | Karin Rybak |
| Gilles O'Hara | Alessandro Politano | Samir Said |
| Kostas Oikonomou | Zdravka Poljakovic | Tamotsu Sakamoto |
| Claudia Olivares | Stewart Pollock | Abraham Salacata |
| Richard Oliver | Jose Polo Garcéa | Adrien Salem |
| Rafael Olvera Ruiz | Holger Poppert | Rafael Salguero Bodes |
| Christoforos Olympios | Maurizio Porcu | Marco A. Saltzman |
| Anna omaszuk-Kazberuk | Antonio Pose Reino | Alessandro Salvioni |
| Joaquín Osca Asensi | Neeraj Prasad | Gregorio Sanchez Vallejo |

| Marcelo Sanmartín Fernández | Adam Sokal | Tian Ming Tu |
| --- | --- | --- |
| Wladmir Faustino Saporito | Yannie Soo Oi Yan | Ype Tuininga |
| Kesari Sarikonda | Rodolfo Sotolongo | Minang Turakhia |
| Taishi Sasaoka | Olga Ferreira de Souza | Samir Turk |
| Hamdi Sati | Jon Arne Sparby | Wayne Turner |
| Irina Savelieva | Jindrich Spinar | Arnljot Tveit |
| Pierre-Jean Scala | David Sprigings | Richard Tytus |
| Peter Schellinger | Alex C. Spyropoulos | C Valadão |
| Carlos Scherr | Dimitrios Stakos | P.F.M.M. van Bergen |
| Lisa Schmitz | Clemens Steinwender | Philippe van de Borne |
| Karl-Heinz Schmitz | Georgios Stergiou | B.J. van den Berg |
| Bettina Schmitz | Ian Stiell | C van der Zwaan |
| Teresa Schnabel | Marcus Stoddard | M. Van Eck |
| Steffen Schnupp | Anastas Stoikov | Peter Vanacker |
| Peter Schoeniger | Witold Streb | Dimo Vasilev |
| Norbert Schön | Ioannis Styliadis | Vasileios Vasilikos |
| Peter Schwimmbeck | Guohai Su | Maxim Vasilyev |
| Clare Seamark | Xi Su | Srikar Veerareddy |
| Greg Searles | Wanda Sudnik | Mario Vega Miño |
| Karl-Heinz Seidl | Kai Sukles | Asok Venkataraman |
| Barry Seidman | Xiaofei Sun | Paolo Verdecchia |
| Jaroslaw Sek | H. Swart | Francesco Versaci |
| Lakshmanan Sekaran | Janko Szavits-Nossan | Ernst Günter Vester |
| Carlo Serrati | Jens Taggeselle | Hubert Vial |
| Neerav Shah | Yuichiro Takagi | Jason Victory |
| Vinay Shah | Amrit Pal Singh Takhar | Alejandro Villamil |
| Anil Shah | Angelika Tamm | Marc Vincent |
| Shujahat Shah | Katsumi Tanaka | Anthony Vlastaris |
| Vijay Kumar Sharma | Tanyanan Tanawuttiwat | Jürgen vom Dahl |
| Louise Shaw | Sherman Tang | Kishor Vora |
| Khalid H. Sheikh | Aylmer Tang | Robert B. Vranian |
| Naruhito Shimizu | Giovanni Tarsi | Paul Wakefield |
| Hideki Shimomura | Tiziana Tassinari | Ningfu Wang |
| Dong-Gu Shin | Ashis Tayal | Mingsheng Wang |
| Eun-Seok Shin | Muzahir Tayebjee | Xinhua Wang |
| Junya Shite | J.M. ten Berg | Feng Wang |
| Gerolamo Sibilio | Dan Tesloianu | Tian Wang |
| Frank Silver | Salem H.K. The | Alberta L. Warner |
| Iveta Sime | Dierk Thomas | Kouki Watanabe |
| Tim A. Simmers | Serge Timsit | Jeanne Wei |
| Narendra Singh | Tetsuya Tobaru | Christian Weimar |
| Peter Siostrzonek | Andrzej R. Tomasik. | Stanislav Weiner |
| Didier Smadja | Mikhail Torosoff | Renate Weinrich |
| David W. Smith | Emmanuel Touze | Ming-Shien Wen |
| Marcelo Snitman | Elina Trendafilova | Marcus Wiemer |
| Dario Sobral Filho | W. Kevin Tsai | Preben Wiggers |
| Hassan Soda | Hung Fat Tse | Andreas Wilke |
| Carl Sofley | Hiroshi Tsutsui | David Williams |

| Marcus L. Williams | Ping Yen Bryan Yan | Ping Zhang |
| --- | --- | --- |
| Bernhard Witzenbichler | Tianlun Yang | Jun Zhang |
| Brian Wong | Jing Yao | Shui Ping Zhao |
| Ka Sing Lawrence Wong | Kuo-Ho Yeh | Yujie Zhao |
| Beata Wozakowska-Kaplon | Wei Hsian Yin | Zhichen Zhao |
| Shulin Wu | Yoto Yotov | Yang Zheng |
| Richard C. Wu | Ralf Zahn | Jing Zhou |
| Silke Wunderlich | Stuart Zarich | Sergio Zimmermann |
| Nell Wyatt | Sergei Zenin | Andrea Zini |
| John (Jack) Wylie | Elisabeth Louise Zeuthen | Steven Zizzo |
| Yong Xu | Huanyi Zhang | Wenxia Zong |
| Xiangdong Xu | Donghui Zhang | L Steven Zukerman |
| Hiroki Yamanoue | Xingwei Zhang |  |
| Takeshi Yamashita |  |  |

**DATA SHARING STATEMENT**

# To ensure independent interpretation of clinical study results, Boehringer Ingelheim grants all external authors access to all relevant material, including participant-level clinical study data, and relevant material as needed by them to fulfill their role and obligations as authors under the ICMJE criteria.

# Furthermore, clinical study documents (eg, study report, study protocol, statistical analysis plan) and participant clinical study data are available to be shared after publication of the primary manuscript in a peer-reviewed journal and if regulatory activities are complete and other criteria met per the BI Policy on Transparency and Publication of Clinical Study Data: https://trials.boehringer-ingelheim.com/

# Prior to providing access, documents will be examined, and, if necessary, redacted and the data will be de-identified, to protect the personal data of study participants and personnel, and to respect the boundaries of the informed consent of the study participants.

# Clinical Study Reports and Related Clinical Documents can also be requested via the link https://trials.boehringer-ingelheim.com/

# All requests will be governed by a Document Sharing Agreement.

# Bona fide, qualified scientific and medical researchers may request access to de-identified, analyzable participant clinical study data with corresponding documentation describing the structure and content of the datasets. Upon approval, and governed by a Data Sharing Agreement, data are shared in a secured data-access system for a limited period of 1 year, which may be extended upon request.

Researchers should use the https://trials.boehringer-ingelheim.com/ link to request access to study data.

**METHODS**

**1.** **Inclusion/Exclusion Criteria**

| **Inclusion** | **Exclusion** |
| --- | --- |
| Age ≥18 years at enrollment (and ≥20 years for Japan)  Male or female patient (or legally acceptable representative) willing and able to provide written informed consent  Newly diagnosed (<3 months prior to baseline visit; and < 4.5 months for Latin America) with nonvalvular AF. Documentation of AF by 12-lead electrocardiogram (ECG), ECG rhythm strip, pacemaker/implantable cardioverter defibrillator electrocardiogram, or Holter ECG (duration of AF episode at least 30 seconds) needed for all enrolled patients  CHA_2_DS_2_-VASc score of ≥1. This requires the presence of at least one of the following risk factors:  a. Congestive heart failure (New York Heart Association Class 2 or greater) or moderate to severe left ventricular systolic dysfunction (eg, left ventricular ejection fraction ≤40%)  b. History of hypertension or systolic blood pressure >160 mm Hg  c. Diabetes mellitus  d. History of stroke, transient ischemic attack, or systemic embolism  e. Vascular disease defined as prior myocardial infarction, peripheral artery disease, complex aortic plaque  f. Age ≥65 years  g. Female  Although AF diagnosis is a baseline requirement, patients are not required to have an ongoing AF episode at the time of entry into this Registry Program | Presence of any mechanical heart valve, or valve disease that is expected to require valve replacement intervention (surgical or nonsurgical) during the course of the assigned registry phase  Received >60 days of VKA treatment in their lifetime prior to the patient’s baseline visit  AF with a generally reversible cause (eg, cardiac surgery, pulmonary embolism, untreated hyperthyroidism)  Life expectancy is expected to be <1 year at the time of potential enrollment as assessed by the investigator  Medical condition other than AF for which chronic use of an oral anticoagulant (eg, a VKA) is indicated  Current participation in any clinical trial of a drug or device  Current participation in an international registry on the use of oral anticoagulation in AF  Patient was enrolled in any other phase of the GLORIA-AF Program  Patient with no further follow-up possible with enrolling investigator during planned study period (such as anticipated relocation) |

**2. Definition of Life-Threatening Bleeds**

Life-threatening bleeding was defined as meeting one or more of the following criteria:

- Symptomatic intracranial bleed
- Reduction in hemoglobin of ≥50 g/l
- Transfusion of ≥4 units of blood or packed cells, associated with hypotension requiring the use of intravenous inotropic agents
- Necessitated surgical intervention
- Fatal bleeding

**3. Further Details of the Imputation Method for Missing Data**

Multiple imputation with chained equations was used to impute missing values for baseline characteristic variables and missing cause of death (White IR, Royston P, Wood AM. Multiple imputation using chained equations: Issues and guidance for practice. Stat Med 2011;30:377-399). The imputation models were constructed based on all collected baseline patient characteristics variables collected in this study including those used in the multivariable analyses and propensity score analysis as well as the important outcomes variables (event/censoring indicators and corresponding cumulative baseline hazard at the time to event/censoring). Twenty imputed datasets were used.

These multiple imputed data sets were then analyzed by using standard procedures for complete data and combining the results from these analyses using Rubin’s rule, except for incidence rates, for which the bootstrap approach was applied to obtain the confidence intervals (Schomaker M, Heumann C. Bootstrap inference when using multiple imputation. Stat Med. 2018;37:2252-2266).

**4. Further Details of the Propensity-Score-Trimmed and Matched Patient Sets**

The propensity scores were calculated for dabigatran vs rivaroxaban, dabigatran vs apixaban, and rivaroxaban vs apixaban; and then restricted set and matched sets for pairs compared were derived.

Propensity scores were calculated within each geographic region to predict the probability of treatment choices according to predefined relevant patient characteristics. Trimming was done separately for each geographic region. The propensity-score-trimmed patient set was obtained by excluding patients who had propensity scores outside the mutual range common to dabigatran and rivaroxaban, or dabigatran and apixaban, with cut-offs at the 1.5th percentile of the propensity distribution for the dabigatran-exposed group, and the 98.5th percentile of the distribution for the rivaroxaban-exposed or apixaban-exposed group. The propensity-score-trimmed patient set for rivaroxaban and apixaban was obtained using cut-offs at the 1.5th percentile of the propensity distribution for the rivaroxaban-exposed group, and the 98.5th percentile of the distribution for the apixaban-exposed group. Excluding these patients from the tails of the propensity score distribution reduces channeling bias and improves the validity of comparisons

The “matched patient set” was generated from within the “trimmed patient set” by matching dabigatran patients to rivaroxaban or apixaban patients and matching rivaroxaban patients to apixaban patients by 1:1 greedy nearest-neighbor matching using calipers equal to 0.2 (standard deviations of the logit of the estimated propensity score). The matching was performed within region.

**5. Further Details of the Additional Covariate Selection Procedure for the Multivariable Cox Regression Model**

A further covariate selection procedure was investigated in the baseline model with the following variables: region, congestive heart failure, left ventricular hypertrophy, systemic embolism, previous stroke/transient ischemic attack/systemic embolism (used for major bleeding analyses only, as not being included in the corresponding set of core variables), prior myocardial infarction (used for major bleeding analyses only, as not being included in the corresponding set of core variables), complex aortic plaque, hypertension, diabetes mellitus (not used for myocardial infarction analyses, as being included in the corresponding set of the core variables), peripheral arterial disease, creatinine clearance, nonsteroidal anti-inflammatory drug use, type of atrial fibrillation, hepatic disease, alcohol abuse, use of drugs associated with higher bleeding risk, as defined in the HAS-BLED score (ie, antiplatelet agent, COX-2 inhibitor, or other nonsteroidal anti-inflammatory drug) (not used for major bleeding analyses, as being included in the corresponding set of core variables), previous oral anticoagulant use (within 3 months), smoking status, and concomitant medication like antipsychotics.

The selection procedure started off with the core variables included and was performed in a forward manner for further covariates listed above. Only the variable that made the largest effect change on the treatment estimate (ie, a variable that relatively changed the treatment effect estimate [hazard ratio] by >10% in comparison with the model from the previous step) was included in the next step model. This step was repeated on the new model until none of the other variables changed the treatment effect estimate by >10% in comparison to the resulting model from the previous step. Generally, the selection procedure was stopped as soon as the number of degrees of freedom for all covariates in the model ×10 exceeds the number of events.

**6. Further Details of Sensitivity Analyses**

The propensity scores were estimated anew with additional covariates (extended PS): concomitant antiplatelet, creatinine clearance, NSAID use, type of AF, and previous OAC. We used the Mantel- Haenszel method summary point estimator of the rate ratio to summarize the rate ratio across strata defined by the deciles of the extended PS and region (insert here reference to Rothman KJ, Greenland S, Lash TL, eds: Modern Epidemiology, third edition. Baltimore: Walters Kluwer, 2008); the rate ratio can be considered as approximation of a hazard ratio.

# TABLES

# Table 1. Variables used in Cox and PS models

| **Variables in both Cox and PS models** | | **Variables in Cox model only** | **Variables in PS model only** |
| --- | --- | --- | --- |
| - Age group - Sex - Hypertension - Diabetes - Previous stroke/transient ischemic attack - Prior non-CNS systemic embolism - Prior myocardial infarction - Presence of complex aortic plaque - Peripheral artery disease | - Abnormal renal function - Abnormal liver function - Prior bleeding - Use of drugs associated with higher bleeding risk, as defined in the HAS-BLED score (i.e., antiplatelet agent, Cox-2 inhibitor or other non-steroidal anti-inflammatory drug) - Congestive heart failure - Left ventricular dysfunction - Alcohol abuse - Smoking - Psychosocial factors/depression | - Concomitant antiplatelet use - Creatinine clearance - Nonsteroidal anti-inflammatory drug use - Type of atrial fibrillation - Previous oral anticoagulant use (within 3 months) | - Coronary artery disease |

CNS – central nervous system; PS – propensity score.

# Table 2. The baseline characteristics of the eligible patients treated with dabigatran, rivaroxaban and apixaban.

| **Characteristics** | **Dabigatran**  **(*N* = 3,839)** | **Rivaroxaban**  **(*N* = 4,015)** | **Apixaban**  **(*N* = 4,505)** |
| --- | --- | --- | --- |
| **Age, years, *n* (%)** |  |  |  |
| <65 | 964 (25.1) | 1,009 (25.1) | 884 (19.6) |
| 65 to <75 | 1,527 (39.8) | 1,527 (38.0) | 1,597 (35.4) |
| ≥75 | 1,348 (35.1) | 1,479 (36.8) | 2,024 (44.9) |
| **Female sex, *n* (%)** | 1,718 (44.8) | 1,766 (44.0) | 2,104 (46.7) |
| **Creatinine clearance, mL/min, *n* (%)** |  |  |  |
| <30 | 43 (1.1) | 61 (1.5) | 97 (2.2) |
| 30 to <50 | 326 (8.5) | 394 ( 9.8) | 608 (13.5) |
| 50 to <80 | 1,284 (33.4) | 1,265 (31.5) | 1,455 (32.3) |
| ≥80 | 1,314 (34.2) | 1,608 (40.0) | 1,581 (35.1) |
| Missing | 872 (22.7) | 687 (17.1) | 764 (17.0) |
| **Type of AF, *n* (%)** |  |  |  |
| Paroxysmal | 2,082 (54.2) | 2,302 (57.3) | 2,614 (58.0) |
| Persistent | 1,309 (34.1) | 1,381 (34.4) | 1,529 (33.9) |
| Permanent | 448 (11.7) | 332 (8.3) | 362 (8.0) |
| **Medical history, *n* (%)** |  |  |  |
| Congestive heart failure | 695 (18.1) | 718 (17.9) | 776 (17.2) |
| History of hypertension | 2,890 (75.3) | 3,040 (75.7) | 3,490 (77.5) |
| Diabetes mellitus | 828 (21.6) | 994 (24.8) | 1,051 (23.3) |
| Previous stroke | 441 (11.5) | 307 (7.6) | 576 (12.8) |
| Coronary artery disease | 511 (13.3) | 715 (17.8) | 874 (19.4) |
| Prior bleeding | 138 (3.6) | 211 (5.3) | 258 (5.7) |
| Alcohol abuse (>8 units/week) | 230 (6.0) | 318 (7.9) | 370 (8.2) |
| Current smoker | 388 (10.1) | 322 (8.0) | 374 (8.3) |
| Past smoker | 937 (24.4) | 1,382 (34.4) | 1,590 (35.3) |
| Previous OAC use within 3 months | 1,699 (44.3) | 2,185 (54.4) | 2,189 (48.6) |
| CHA_2_DS_2_-VASc score, mean (SD) | 3.1 (1.4) | 3.1 (1.5) | 3.4 (1.5) |
| HAS-BLED, mean (SD) | 1.2 (0.8) | 1.2 (0.8) | 1.4 (0.9) |
| **Chronic concomitant medications, *n* (%)** |  |  |  |
| Antiplatelet | 508 (13.2) | 765 (19.1) | 930 (20.6) |
| Drugs with higher bleeding risk (HAS-BLED)* | 569 (14.8) | 885 (22.0) | 1077 (23.9) |
| **Region, *n* (%)** |  |  |  |
| Asia | 930 (24.2) | 384 ( 9.6) | 384 ( 8.5) |
| Europe | 2,066 (53.8) | 2,008 (50.0) | 2,197 (48.8) |
| North America | 432 (11.3) | 1,338 (33.3) | 1,753 (38.9) |
| Latin America | 411 (10.7) | 285 (7.1) | 171 (3.8) |
| **Treatment dose, *n* (%)** |  |  |  |
|  | 150mg bid: 2005 (52.2) | 10mg OD: 112 (2.8) | 5mg bid: 3582 (79.5) |
|  | 110mg bid: 1728 (45.0) | 15mg OD: 856 (21.3) | 2.5mg bid: 880 (19.5) |
|  | 75mg bid: 55 (1.4) | 20mg OD: 2992 (74.5) | Other dose: 43 (1.0) |
|  | Other dose: 51 (1.3) | Other dose: 55 (1.4) |  |

* Concomitant use of drugs associated with higher bleeding risk (ie, antiplatelet agent, Cox-2 inhibitor or other non-steroidal anti-inflammatory drug).

AF – atrial fibrillation; OAC – oral anticoagulant; HAS-BLED – Hypertension, Abnormal liver/renal function, Stroke history, Bleeding history or predisposition, Labile international normalized ratio (INR), elderly (age >65 years), Drug/alcohol usage; CHA_2_DS_2_-VASc – congestive heart failure, hypertension, age ≥75 years, diabetes mellitus, stroke/ transient ischemic attack/systemic embolism, vascular disease, age 65 to 74 years, sex category (female); SD – standard deviation; OD – once daily; bid – twice daily; mg – milligram.
