# Supplementary figures and images for: Comparative effectiveness and safety of non-vitamin K antagonists for atrial fibrillation in clinical practice: GLORIA-AF Registry
Source: Clin Res Cardiol. 2022 Mar 16;111(5):560–73. doi: 10.1007/s00392-022-01996-2 (PMC9054878; doi:10.1007/s00392-022-01996-2)

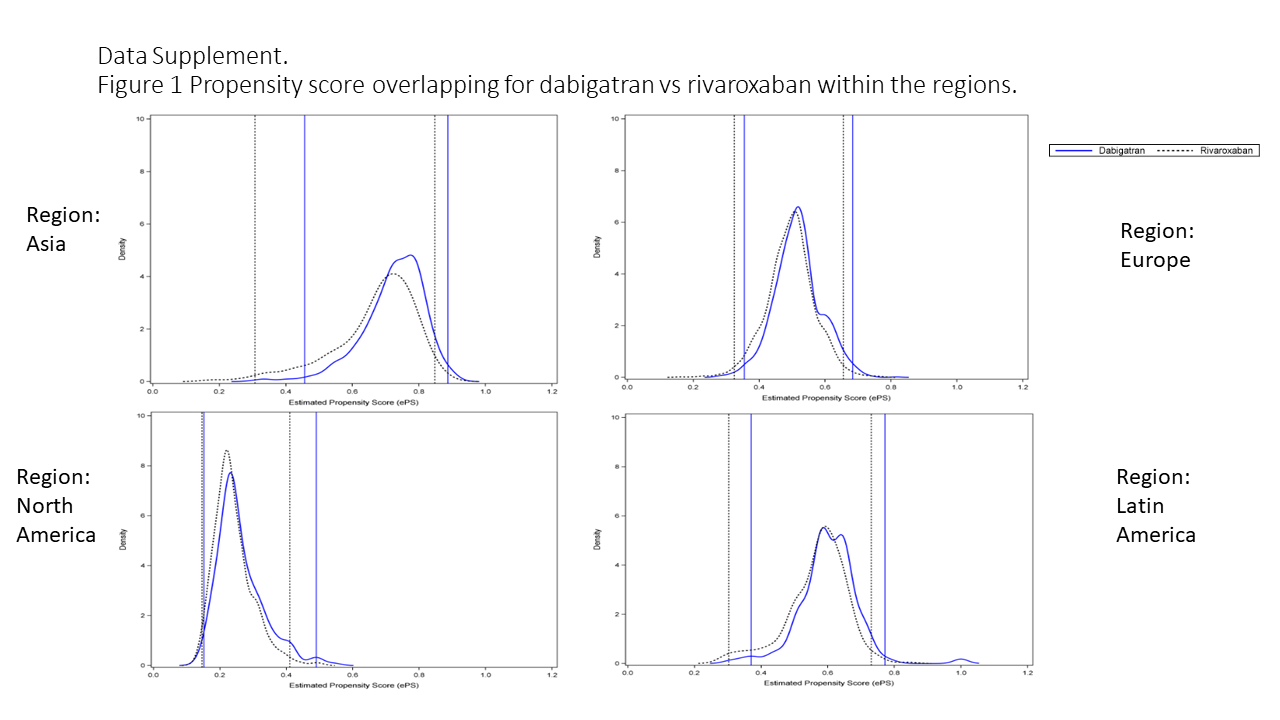

Supplement: Supplementary file 2 — Supplementary file2 (TIFF 167 KB) [file 392_2022_1996_MOESM2_ESM.tif]

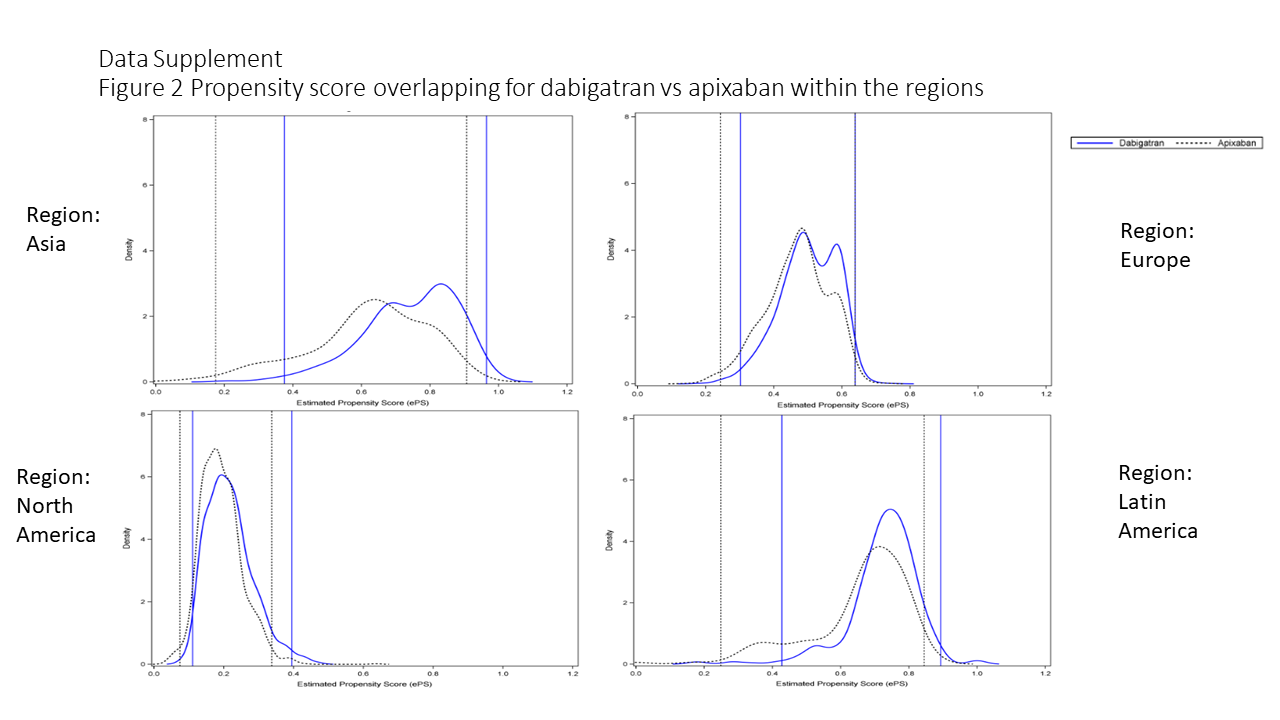

Supplement: Supplementary file 3 — Supplementary file3 (TIFF 168 KB) [file 392_2022_1996_MOESM3_ESM.tif]

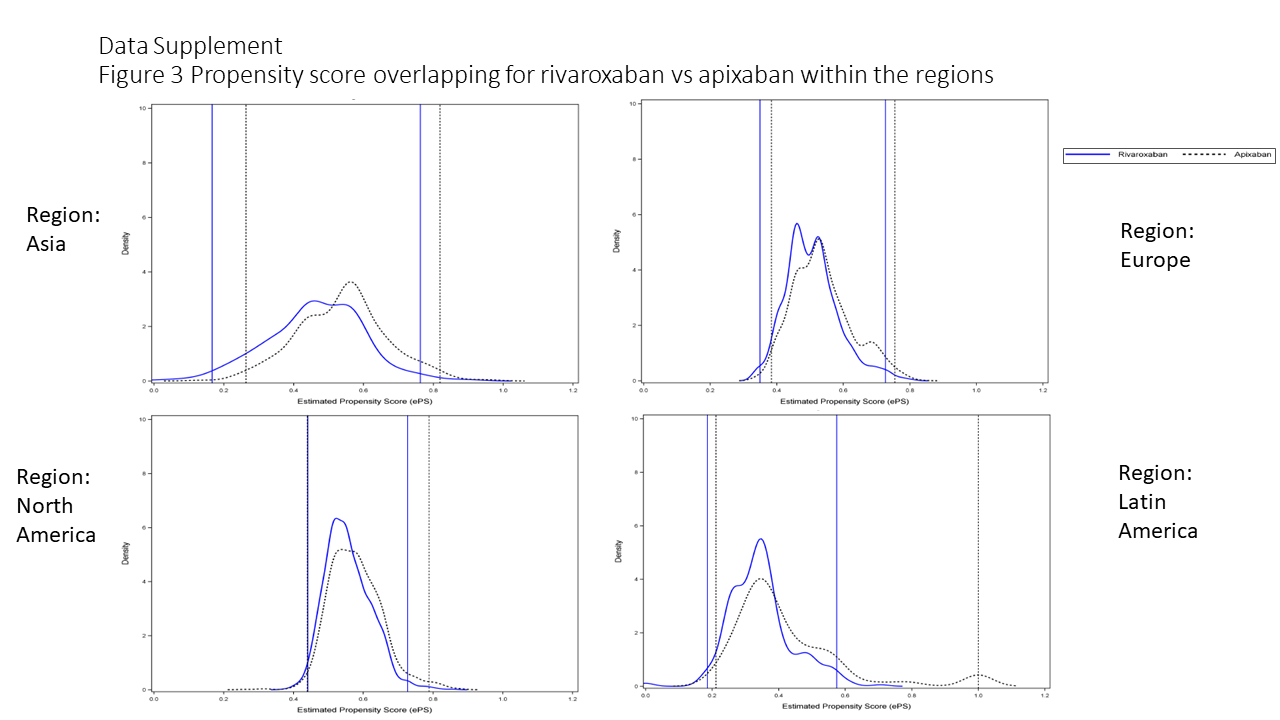

Supplement: Supplementary file 4 — Supplementary file3 (TIFF 161 KB) [file 392_2022_1996_MOESM4_ESM.tif]
